# How Does the L884P Mutation Confer Resistance to Type-II Inhibitors of JAK2 Kinase: A Comprehensive Molecular Modeling Study

**DOI:** 10.1038/s41598-017-09586-3

**Published:** 2017-08-22

**Authors:** Xiaotian Kong, Huiyong Sun, Peichen Pan, Dan Li, Feng Zhu, Shan Chang, Lei Xu, Youyong Li, Tingjun Hou

**Affiliations:** 1 Institute of Functional Nano and Soft Materials (FUNSOM), Soochow University, Suzhou, Jiangsu 215123 P. R. China; 2 College of Pharmaceutical Sciences, Zhejiang University, Hangzhou, Zhejiang, 310058 P. R. China; 3 Institute of Bioinformatics and Medical Engineering, School of Electrical and Information Engineering, Jiangsu University of Technology, Changzhou, 213001 China

## Abstract

Janus kinase 2 (JAK2) has been regarded as an essential target for the treatment of myeloproliferative neoplasms (MPNs). BBT594 and CHZ868, Type-II inhibitors of JAK2, illustrate satisfactory efficacy in preclinical MPNs and acute lymphoblastic leukemia (ALL) models. However, the L884P mutation of JAK2 abrogates the suppressive effects of BBT594 and CHZ868. In this study, conventional molecular dynamics (MD) simulations, umbrella sampling (US) simulations and MM/GBSA free energy calculations were employed to explore how the L884P mutation affects the binding of BBT594 and CHZ868 to JAK2 and uncover the resistance mechanism induced by the L884P mutation. The results provided by the US and MD simulations illustrate that the L884P mutation enhances the flexibility of the allosteric pocket and alters their conformations, which amplify the conformational entropy change (−TΔ*S*) and weaken the interactions between the inhibitors and target. Additionally, the structural analyses of BBT594 and CHZ868 in complex with the WT JAK2 illustrate that the drug tail with strong electronegativity and small size located in the allosteric pocket of JAK2 may enhance anti-resistance capability. In summary, our results highlight that both of the changes of the conformational entropies and enthalpies contribute to the L884P-induced resistance in the binding of two Type-II inhibitors into JAK2 kinase.

## Introduction

**Figure 1:**
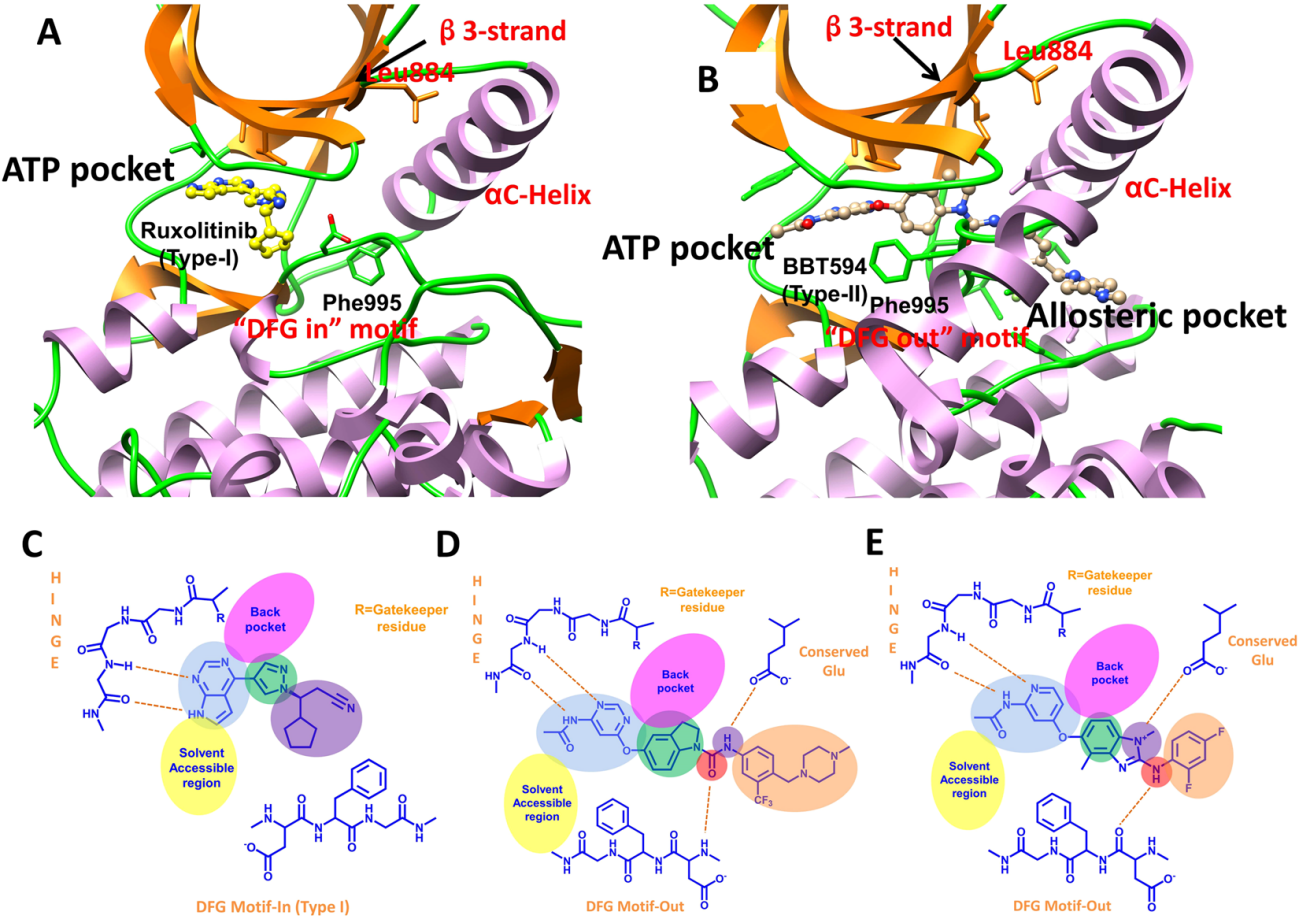
Type-I inhibitor Ruxolitinib bound to JAK2 with the DFG-in conformation (PDB code: 4U5J, panel A), and Type-II inhibitor BBT594 bound to JAK2 with the DFG-out conformation (PDB entry: 3UGC, panel B). The 2D-interactions between JAK2 and Ruxolitinib, BBT594, and CHZ868 are shown in panels C~E.

Janus kinase 2 (JAK2) is a non-receptor tyrosine kinase associated with the cytoplasmic domain of cytokine receptors^1^ and plays important roles in cytokine signaling via the JAK-STAT (signal transducers and activators of transcription) signaling pathway^2–4^. Genetic and functional studies have identified somatic JAK2^V617F^ mutation and other mutation alleles that activate the JAK-STAT signaling in most patients with myeloproliferative neoplasms (MPNs)^5–11^. The therapeutic importance of JAK2 accelerates the development of its inhibitors, and a number of ATP competitive (Type-I) inhibitors with good efficacy have even been pushed into preclinical and clinical stages^12–16^, such as the FDA approved JAK2 inhibitor Ruxolitinib (Fig. 1A) for the treatment of myelofibrosis and hydroxyurea-resistant polycythemia vera (PV)^17–21^.

JAK2 inhibitors have two general categories: Type-I and Type-II. Type-I inhibitors occupy the ATP-binding pocket in the active conformation (DFG-in), and Type-II inhibitors occupy not only the ATP-binding pocket in the inactive conformation (DFG-out) but also an adjacent allosteric pocket that is available when JAK2 is inactive. A large number of Type-I JAK2 inhibitors have been reported, but most of them cannot achieve good JAK2 selectivity because the sequences and structures of the ATP binding sites of the JAK isoforms are quite similar. In contrast, it may be easier to design JAK2 selective Type-II inhibitors because a less conserved allosteric pocket adjacent to the ATP-binding pocket can form direct interaction with Type-II JAK2 inhibitors. Although all JAK2 inhibitors in clinical pipeline are Type-I inhibitors, some progresses on the discovery of Type-II JAK2 inhibitors have still been made in recent years. As two representative Type-II JAK2 inhibitors, BBT594 and CHZ868 (Fig. 1B) show good potency and selectivity toward JAK2 (BBT594: IC_50_ = 0.99; CHZ868: IC_50_ = 0.11 uM, Table 1), and are also effective towards several hematological malignancies that are always refractory to Type-I JAK2 drugs^22–26^. Andraos and colleagues identified that, by stabilizing JAK2 in an inactive conformation, BBT594 could blunt the phosphorylation of JAK2 A-loop and STAT5 in several myeloid cells, such as Ba/F3 and MHH-CALL-4 cells^22^. Soon after, two studies reported by Meyer *et al*. and Wu *et al*. characterized another Type-II JAK2 inhibitor CHZ868, which is more effective than BBT594 and exhibits striking efficacy in JAK2-dependent MPNs and B cell acute lymphoblastic leukemia (B-ALL) models^26, 27^. Moreover, both BBT594 and CHZ868 are more potent than most Type-I inhibitors in inducing the apoptosis of mutant cells, such as JAK2 V617F and CRLF2-JAK2 R683G^25^.

**Table 1:** PMF depth (ΔW_PMF_) of the two Type-II inhibitors in complex with the WT and L884P JAK2s calculated by the US simulations (kcal/mol).

	WT/BBT594	L884P/BBT594	WT/CHZ868	L884P/CHZ868
PMF_7 ns	20.47^a^ ± 0.10^b^	14.99 ± 0.16	23.78 ± 0.14	21.91 ± 0.23
PMF_8 ns	19.58 ± 0.13	16.78 ± 0.12	23.67 ± 0.10	21.97 ± 0.28
PMF_9 ns	19.60 ± 0.16	18.22 ± 0.14	23.53 ± 0.11	21.71 ± 0.11
PMF_10 ns	19.80 ± 0.19	16.75 ± 0.14	23. 63 ± 0.15	20.95 ± 0.26
PMF_Average (4 ns)	19.84 ± 0.13^c^	16.68 ± 0.13	23.65 ± 0.12	21.79 ± 0.20
IC_50_ (uM)	0.99	10.89	0.11	0.44
Δ*G*_bind_^d^	−25.30 ± 0.94	−21.70 ± 1.09	−29.10 ± 1.88	−27.50 ± 1.38

^a^The PMF value was estimated by averaging the bins across 18~20 Å of the RC. ^b^The standard deviation of each 1 ns US simulation (7~10 ns) was estimated based on the bins across 18.5~20 Å of the RC. ^c^The total standard deviations were estimated from the PMF values of the 7–10 ns US simulations. ^d^Binding free energy.

Similar to other kinases, the emergence of resistance mutations, which usually occur in the conserved ATP binding pocket of JAK2 (Fig. 1A and C), significantly attenuates the therapeutic efficiency of JAK2 inhibitors^28–33^. In Ba/F3-CRLF2 cells harboring JAK2 R683G/L884P, the L884P mutation in JAK2 remarkably attenuates the suppressive effects of Type-II inhibitors of JAK2^34^. The R683G mutation localized near the JH2-JH1 interface is supposed to enhance the resistance of the L884P mutation in JAK2 JH1 by destabilizing the JH2-JH1 auto inhibitory interaction^35^. The increases of IC_50_ induced by the L884P mutation are 11- and 4-fold for BBT594 and CHZ868, respectively (Table 1)^25, 26^. Based on the crystal structure of the JAK2/BBT594 complex, it is hypothesized that the mutation of Leu884 to Pro884, located at the end of the β3-strand, can obstruct the key protein-ligand and residue-residue interactions between BBT594 and the binding pocket, which destabilizes the P-loop, β3-strand and αC-helix regions of JAK2^26, 27^. However, the above explanation is relatively ambiguous, and therefore, in this study, conventional molecular dynamics (MD) simulations, enhanced sampling simulations (umbrella sampling, US), and MM/GBSA binding free energy calculations and decompositions were carried out to elucidate the drug resistance mechanism caused by the L884P mutation in JAK2 toward two Type-II inhibitors (BBT594 and CHZ868). We try to understand the impact of the L884P mutation on the flexibility and dynamics of the essential parts of JAK2 to drugs binding, such as β3-strand and αC-helix, and identify the key residue-residue and protein-ligand interactions along the dissociation pathways of BBT594 and CHZ868 from the WT and L884P mutated JAK2s. Then, conformational entropy calculation combined with RMSF and RMSD analysis were carried out to explore the difference of the conformational change between the WT and the L884P mutated systems. Meanwhile, the key protein-ligand interactions related to drug resistance were quantitatively highlighted by MM/GBSA per-residue energy decomposition. We expect that the comprehensive analyses can guide and pave the way for the design of novel JAK2 inhibitors with improved capability to combat drug resistance.

## Results and Discussion

### Favorable Unbinding Pathway for Type-II Kinase Inhibitors

Before analyzing the drug resistance mechanisms of the two inhibitors (BBT594 and CHZ868), we first checked the convergence of the simulated systems. Then the favorable unbinding pathway for each system was determined by choosing the minimized energy pathway from the ATP channel and allosteric channel.

#### Convergence of the Simulated Systems

In order to acquire optimum configurations for US simulations, 30 ns conventional MD simulations were first carried out for each system. As illustrated in Figures S1 and S2, the low RMSDs of the protein-ligand complexes, as well as the protein (active site) and ligand individually, indicate that all the studied systems achieve stability over the equilibrated 2~30 ns conventional MD simulations. (RMSDS < 2.0 Å on average) Hence, the last snapshot of the MD trajectory for each system was used as the initial structure for the following US simulations.

To guarantee the sampling convergence of the US simulations, 10 ns US simulations were performed for each window of all the systems (WT/BBT594, L884P/BBT594, WT/CHZ868, and L884P/CHZ868) along the allosteric or the ATP unbinding pathway, where the convergence of each PMF curve was checked after each nanosecond of the US simulations. As shown in Figures S3 and S4, all the systems converged after ~6 ns US simulations (6~7, 7~8, 8~9 and 9~10 ns), and thus the PMF curves were computed based on the last 4 ns US samples (6~10 ns, PMF values shown in Table 1 were averaged from 18.5~20 Å of the RC for each direction).

#### Allosteric Channel Is the Favorable Unbinding Pathway for Type-II Inhibitors

**Figure 2:**
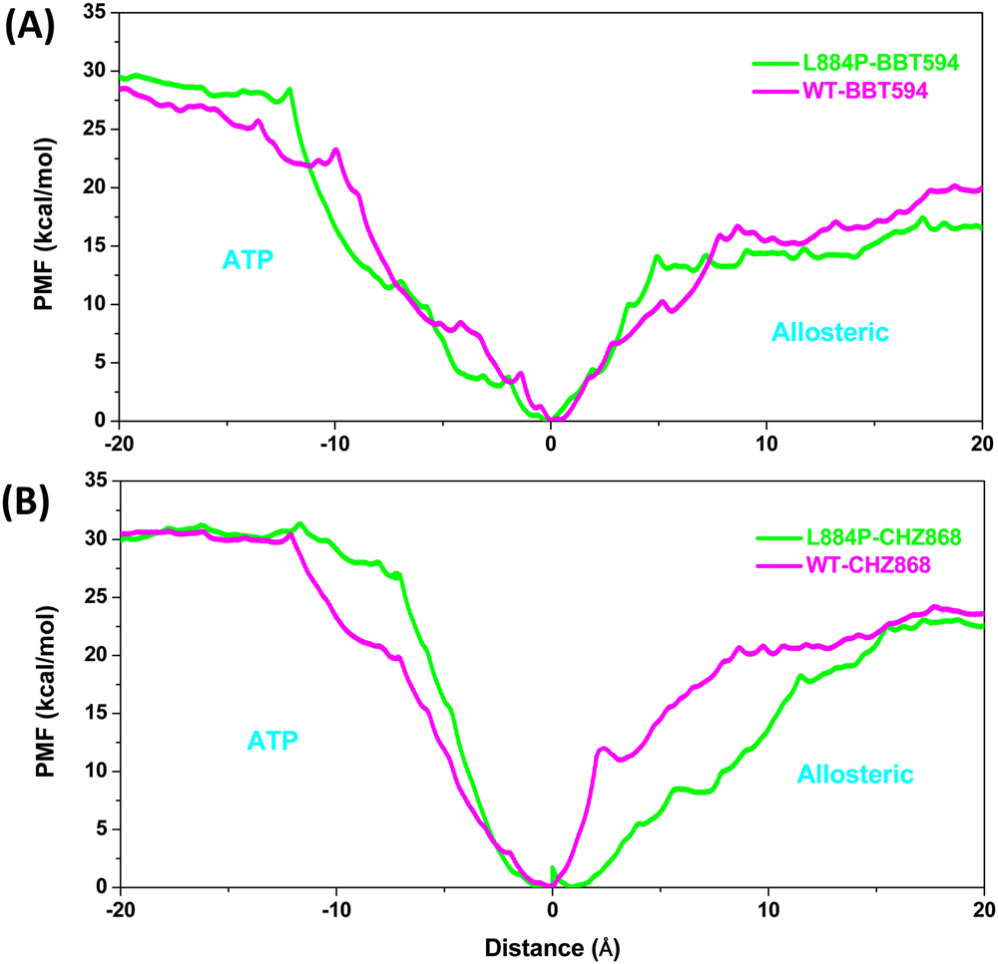
Comparison of the PMF curves for the allosteric and the ATP dissociation pathways of (**A**) WT/BBT594 (magenta) and L884P/BBT594 (green), and (**B**) WT/CHZ868 (magenta) and L884P/CHZ868 (green).

As been discussed above, Type-II inhibitors can occupy both the ATP-binding pocket and the allosteric pocket of kinases, and therefore it is challenging to determine which unbinding pathway is favorable for the dissociation of Type-II inhibitors. Therefore, we performed US simulations for both directions (ATP pocket direction and allosteric pocket direction) in order to determine the pathway that is more favorable for the dissociation of Type-II inhibitors. By connecting the PMF curves of the two directions for all the investigated systems (Fig. 2), it is found that the PMF curves derived from the allosteric pathway are always lower than those derived from the ATP pathway, which is consistent with our previous conclusion that the allosteric pathway is more favorable for the dissociation of two Type-II inhibitors of kinase^36^.

### Drug Resistance Mechanisms Characterized by US simulations

**Figure 3:**
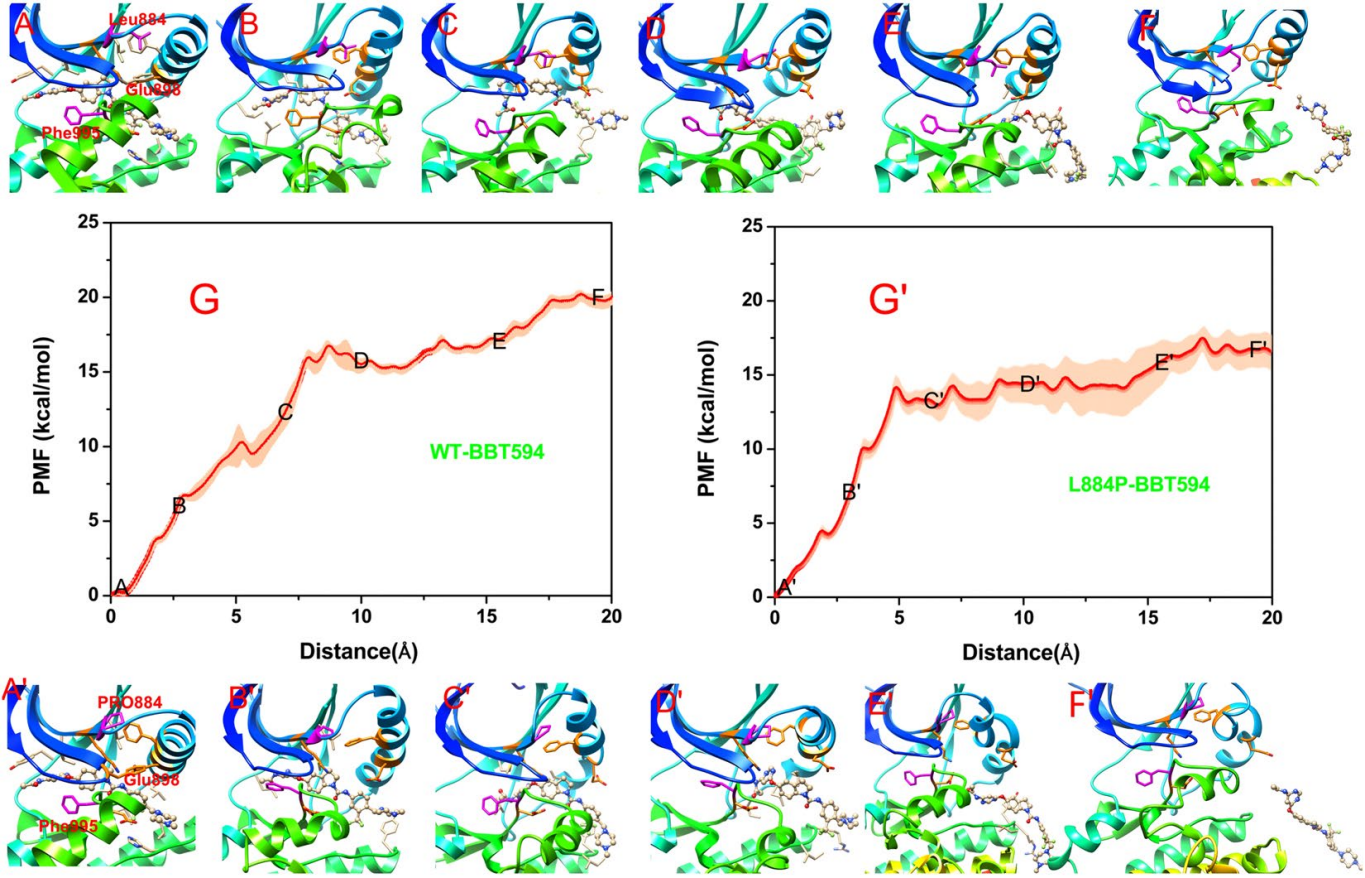
Unbinding processes of Type-II inhibitor BBT594 dissociating from the binding sites of the WT (panels A~F) and L884P (panels A’~F’) JAK2 along the allosteric channel. (the individual pictures of Fig. 3A~F and 3A’~F’ correspond to in Figure S5A~F and S5A’~S5F’ in Figure S5 of supplementary information).

**Figure 4:**
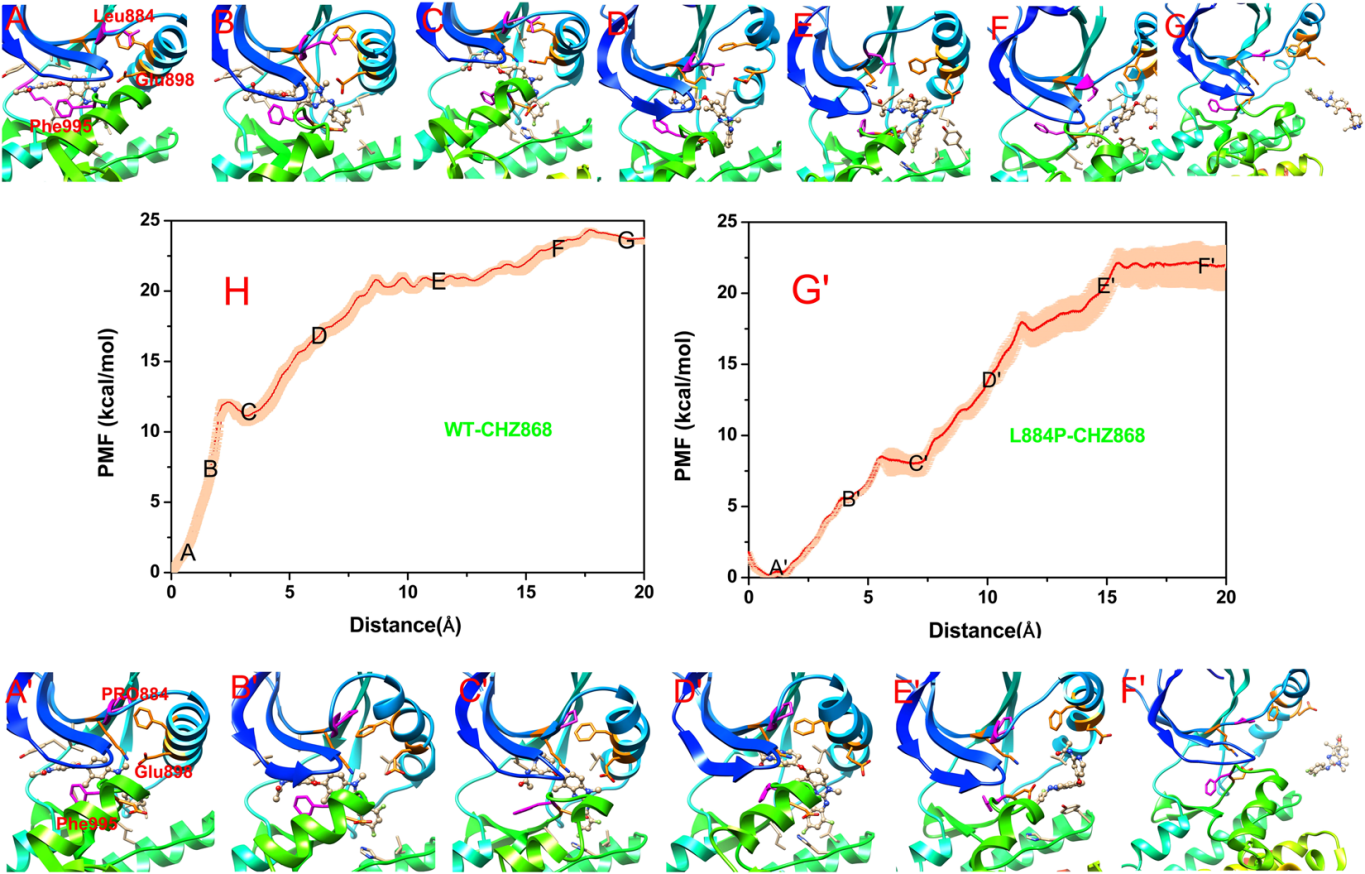
Unbinding processes of Type-II inhibitor CHZ868 dissociating from the binding sites of the WT (panels A~G) and L884P (panels A’~F’) JAK2 along the allosteric channel. (the individual pictures of Figure 4A~G and 4A’~F’ correspond to Figures S6A~G and S6A’~S6F’ in Figure S5 of supplementary information).

As shown in Figs 3G and 4H, the energy profiles of WT/BBT594 and WT/CHZ868 are relatively higher than those of the corresponding mutated systems (L884P/BBT594, Fig. 3G’; L884P/CHZ868, Fig. 4G’). As shown in Table 1, the binding affinities (PMF depth, ΔW_PMF_) are 19.8, 16.7, 23.7 and 21.8 kcal/mol for WT/BBT594, L884P/BBT594, WT/CHZ868 and L884P/CHZ868, respectively, suggesting that the Type-II inhibitors can form relatively tighter interactions with the WT target than with the L884P mutant. That is to say, the L884P mutation can induce resistance to both BBT594 and CHZ868, but it has slightly more impact on BBT594, which is qualitatively consistent with the experimental data^25, 26^. The drug resistance mechanisms are detailed in the following section.

#### Comparison of the Reaction Coordinates (RCs) for the WT/BBT594 and L884P/BBT594 systems

As shown in Fig. 3 (Figure S5), when BBT594 horizontally escapes from the allosteric channel, there is a steep upgrading stage of the PMF (0~5 Å of the RC, Fig. 3G) because of the breakage of the H-bonds between the BBT594 amino-pyrimidine fragment and the backbone-CO/NH of Leu932, where the ligand remains in its original conformation (Figs 3B or S5B). During the stage of 5.0~8.5 Å of the RC (Fig. 3G), the H-bond interactions between the urea-CO/NH of BBT594 and Asp994/Glu898 attenuate gradually (Figs 3C or S5C), and meanwhile, the 2,3-dihydro-1H-indoleand amino-pyrimidine fragment successively approaches to the residues (Asp994 and Phe995) in the DFG motif and some hydrophobic residues (Ile901 and Leu902) in the αC-helix, where the αC-helix moves upward and is forced to make way for the bulky drug. Due to the high strain energy, the backbone of the drug, soon afterwards, collapses and rotates to a larger space to relax the high energy state which corresponds to the decrease of the PMF curve (Figs 3D or S5D, 8.5~11.5 Å of the RC). Finally, BBT594 struggles to shake off the absorption of the A-loop residues (11.5~18.5 Å of the RC, Figs 3E or S5E) and totally dissociates from the target (point F in Fig. 3G). Compared with the PMF curve of WT/BBT594, the PMF profile of L884P/BBT594 exhibits relatively lower values. As displayed in Fig. 3G’, BBT594 in the L884P JAK2 breaks away from the pocket with fewer obstacles, which, according to Fig. 3A’~E’ (Figure S5A’~E’), may be attributed to the conformational change of the allosteric channel induced by the mutation of Leu884 to Pro884. First, the H-bond interactions between BBT594 and some residues (such as Leu932, Glu898 and Asp 994) of the L884P JAK2 are all impaired quickly, thus the L884P system exhibits slightly steeper upgrading PMF curve than WT system(0~5 Å of the RC, Figs 3B’ or S5B’). It is followed by the nearly flat region of the PMF curve (5~14 Å of RC), where the drug constantly adjusts the posture to accommodate itself in the allosteric pocket (Fig. 3C’ and D’, Figure S5C’ and D’), and then completely dissociates from the target (Fig. 3E’ and F’, Figure S5E’ and F’). The whole process seems much smoother than WT, which can be explained by the fewer barriers along the allosteric channel, e.g., the steric hindrance from the αC-helix, DFG motif and A-loop. Based on the above comparison (Figure 3B~E *versus* Fig. 3B’~3E’, Figure S5B~E *versus* Figure S5B’~E’), we can observe that the key secondary structures of the allosteric pocket (αC-helix, DFG motif and β-strand) in the L884P mutant exhibit more flexible behavior than those in WT, which can permit timely positional adjustments to facilitate the unbinding process of BBT594 from the mutant.

In the initial structure of the WT JAK2 shown in Figs 3A or S5A, Leu884, located in the β3-strand of the N-terminal lobe, interacts with Phe895 directly, which is important to stabilize the position of the αC-helix. In addition to the salt bridge with Glu898 in the conserved αC-helix, Lys882 in the β3-strand also forms H-bonds with Gly996 and Phe995 (DFG-out motif). In this interaction network, BBT594 appears to be tightly trapped into a narrow gorge of the binding pocket. However, in the L884P/BBT594 system (Figs 3A’ or S5A’), the transformation of leucine to proline interdicts these interactions, leading to the improved flexibility of the β3-strand and αC-helix and amplifying the space of the allosteric pocket. Thus, it can be concluded that the capacious dissociation channel and the quickly vibrational entrance of the mutated target have negative influence on the binding of BBT594.

#### Comparison of the Reaction Coordinates (RCs) for the WT/CHZ868 and L884P/CHZ868 systems

Compared with BBT594, CHZ868 exhibits better overall efficacy especially in the aspects of its higher inhibitory activity to JAK2 and the relatively good anti-resistance capability to the L884P mutant. The most obvious difference between the WT/CHZ868 and WT/BBT594 systems is the vertical flip of CHZ868 in the WT JAK2, where the amino-pyridine moiety goes firstly out of the pocket (Figures 3B (S5B)~E (S5E), 3.2~16 Å of Fig. 3G *versus* Figures 4B (S6B)~F (S6B), 2.3~17 Å of Fig. 4H). Although the activity difference of CHZ868 in the L884P and WT JAK2s is not obvious, different shape of PMF curve between WT JAK2/CHZ868 and L884P JAK2/CHZ868 is still observed (Fig. 4H *versus* Fig. 4G’). At the beginning of the dissociation event, the PMF curve of WT/CHZ868 rises quickly to overcome the energetic barriers, whereas that of the L884P mutant is relatively moderate (Figs 4B (S6B), 0~1.8 Å of Fig. 4H *versus* Figs 4B’(S6B’), 0~5.6 Å of Fig. 4G’). After that, the ligand in the WT JAK2 adjusts its posture quickly, along with the collapse of the interaction network in the allosteric pocket, to release the too high strain energy, where the PMF curve goes downhill as well (1.8~3.2 Å of RC in Fig. 4H). However, there is no obvious downward phenomenon of the PMF curve in the corresponding position of the L884P mutant. It may be explained by the difference of the initial structures of the WT/CHZ868 and L884P/CHZ868 complexes, where CHZ868 in the WT JAK2 is tightly fettered in the pocket by more intricate interactions between the β-strand, DFG motif and αC-helix, such as the extra H-bond interaction between Lys999 (DFG-in motif) and Asp894 (αC-helix) (Figs 4B or S6B). Whereas, slightly impaired interactions are found to CHZ868 in the allosteric pocket of the L884P JAK2, which may lead to the more smooth PMF curve as shown in Fig. 4G’.

As for the following dissociation process, the PMF curve of L884P/CHZ868 is relatively steeper than that of WT/CHZ868 system. (Fig. 4C~F *versus* Fig. 4C’~E’, Figure S6C~F *versus* Figure S6C’~E’) It can be explained that the remaining interactions of L884P/CHZ868 system are quickly destroyed due to the instability of its allosteric pocket, while for WT/CHZ868 system the dissociation process is accompanied with the energy release derived from the protein-ligand accommodation. However, on the whole, the PMF curve of WT/CHZ868 system is slightly higher than that of L884P/CHZ868. According to the US simulations, changes of conformation and interactions both contribute to drug resistance, which will be quantitatively confirmed by the entropy analysis and enthalpy calculations in the following section.

### Contribution of Conformational Entropy to Drug Resistance

**Figure 5:**
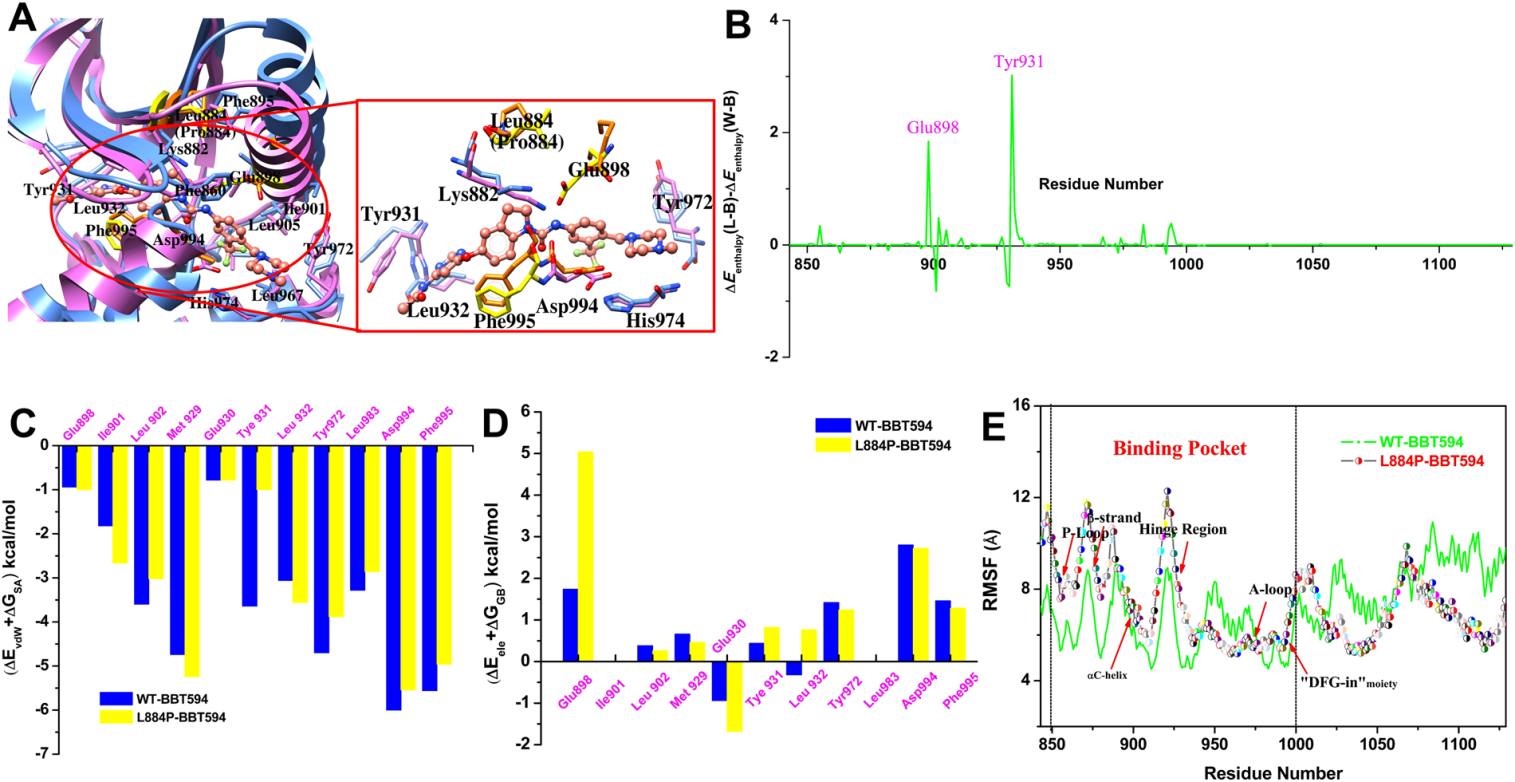
Comparison of the structures of the WT (magenta) JAK2/BBT594 and L884P (blue) JAK2/BBT594 complexes (panel A, key residue in the WT or L884P JAK2 is colored in yellow or orange). Differences of the total interactions (enthalpies) for the WT and L884P JAK2 complexes are illustrated in panel B. Comparison of the non-polar and the polar part contributions for the WT (blue) and L884P (yellow) JAK2 complexes are illustrated in panels C and D. Comparison of the RMSFs of the WT (green) and L884P (colorful)/BBT594 complexes is shown in panel E. (the individual pictures of Fig. 5A~E correspond to Figure S7A~E in Figure S7 of supplementary information).

When receptor-ligand binding events occur, the structures of the receptor and ligand may need large-scale conformational change to accommodate with each other (the so called induced-fit phenomenon). As shown in Table 2, the conformational entropy change (−T∆*S*) for the binding of BBT594 to the L884P JAK2 is slightly larger than that for the binding of BBT594 to the WT JAK2 (26.7 *versus* 27.9 kcal/mol), while the entropy change is much smaller for CHZ868 (25.2 *and* 25.9 kcal/mol for the WT and L884P binding, respectively). We can observe from Figure S2 that the bulky BBT594 ligand is more fluctuant in the binding site than CHZ868. And the RMSDs of BBT594 in L884P/JAK2 system are larger than that in WT/JAK2 system. As for CHZ868 ligand, its flexibilities in WT/JAK2 and L884P/JAK2 are nearly identical. Moreover, the comparison of the root-mean-square fluctuations (RMSFs) between the WT and L884P systems was conducted to explore the conformational difference (WT/BBT594 *versus* L884P/BBT594 and WT/CHZ868 *versus* L884P/CHZ868). To be more specific, as illustrated in Figs 5E (S7E) and 6E (S8E), the residues of the P-loop (857~862) and hinge region (929~933) in the ATP-binding pocket, as well as the residues surrounding the allosteric pocket (879~884 of the β-strand, 993~1000 of the DFG motif, 972~978 of the A-loop and 889~903 of the αC-helix), in the mutated JAK2 exhibit amplified fluctuations over those in the WT JAK2. The higher RMSFs imply larger conformational changes of the binding pockets of the mutated systems compared with those of the WT systems, which is consistent with the results of the conformational entropy change shown in Table 2. That is to say, the loss of the interactions between Leu884 and the αC-helix Phe895, as well as the P-loop Phe860, impairs the stability of the αC-helix, P-loop and DFG-in motif in the mutated JAK2. Moreover, the weak effect of the mutated site (L884P) in the CHZ868/JAK2 system for the conformational entropy change, illustrated by RMSDs and RMSFs analyses, may be explained by the smaller size of CHZ868 and stronger interaction with the protein.

**Table 2:** MM/GBSA binding free energies and the corresponding energetic components of the two Type-II inhibitors in complex with the WT and L884P JAK2s (kcal/mol).

Name	WT/BBT594	L884P/BBT594	WT/CH868	L884P/CHZ868
Δ*E*_ele_^*a*^	−19.17 ± 0.93	−18.67 ± 0.97	−25.82 ± 0.47	−23.79 ± 0.25
Δ*E*_vdW_^*b*^	−72.92 ± 0.28	−71.69 ± 0.52	−63.63 ± 0.63	−62.57 ± 0.73
Δ*G*_GB_^*c*^	46.26 ± 0.73	47.03 ± 0.78	40.36 ± 0.22	38.12 ± 0.16
Δ*G*_SA_^*d*^	−6.19 ± 0.02	−6.25 ± 0.04	−5.18 ± 0.02	−5.16 ± 0.02
∆*E*_non-polar_^*e*^	−79.11 ± 0.28	−77.95 ± 0.52	−68.81 ± 0.63	−67.73 ± 0.73
∆*E*_polar_^*f*^	27.09 ± 0.93	28.36 ± 0.97	14.54 ± 0.47	14.33 ± 0.25
∆*E*_enthalpy_^*g*^	−52.10 ± 0.65	−49.60 ± 0.74	−54.27 ± 0.66	−53.41 ± 0.61
−T∆*S* ^*h*^	26.70 ± 1.24	27.90 ± 1.45	25.20 ± 3.11	25.90 ± 2.16
Δ*G*_bind_^*i*^	−25.30 ± 0.94	−21.70 ± 1.09	−29.10 ± 1.88	−27.50 ± 1.38

^a^Electrostatic interaction. ^b^van der Waals interaction. ^c^Polar contribution of the solvation effect. ^d^Non-polar contribution of solvation effect. ^e^Non-polar interaction. ^f^Polar interaction. ^g^Enthalpic contribution. Standard deviations were estimated based on five blocks. ^h^Entropic contribution. Standard deviations were estimated based on five blocks (Table S1). ^i^Binding free energy. Standard deviations were estimated based on the average standard deviations of enthalpic and entropic contributions.

**Figure 6:**
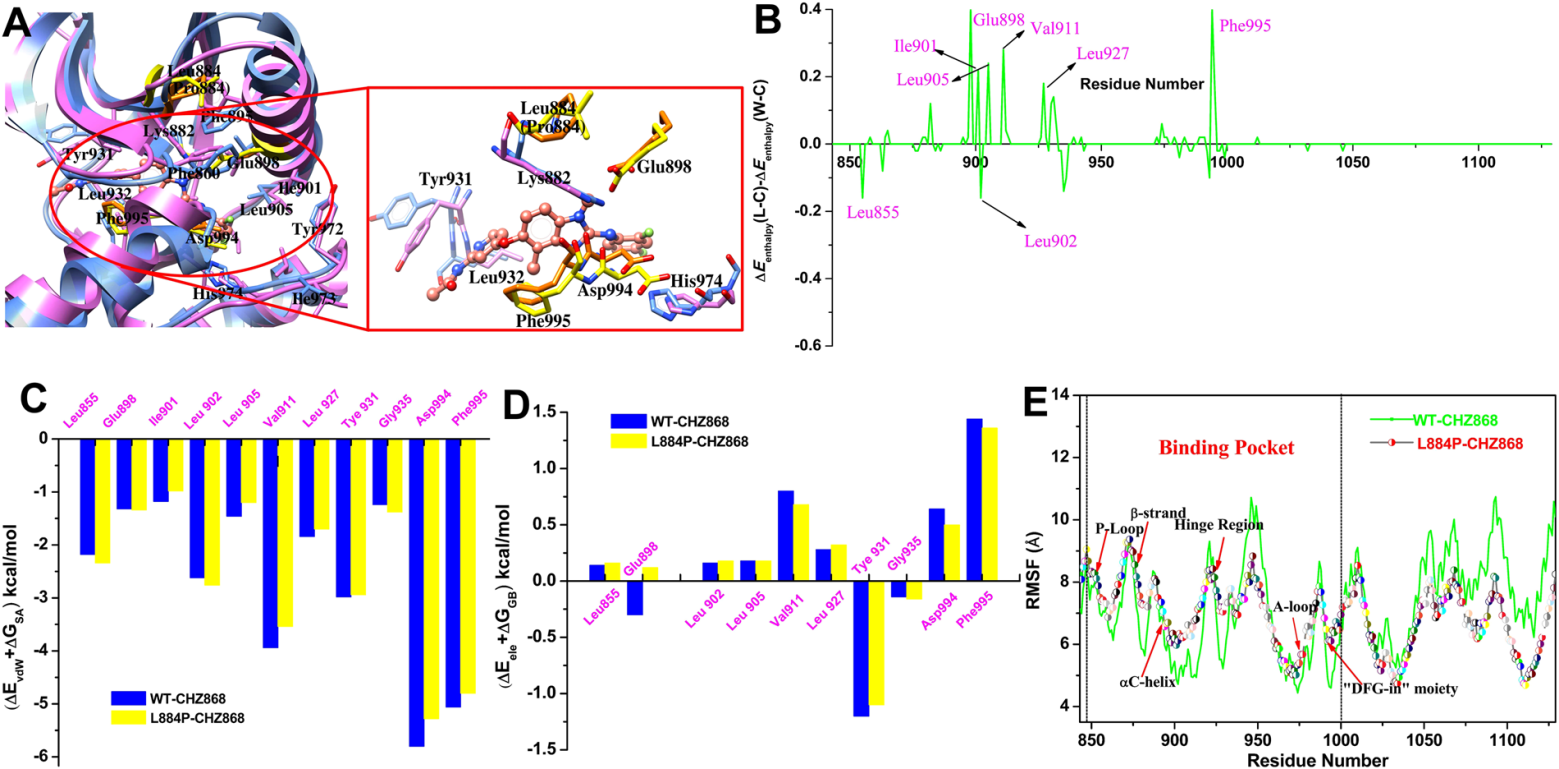
Comparison of the structures of the WT (magenta) JAK2/CHZ868 and L884P (blue) JAK2/CHZ868 complexes (panel A, key residue in the WT or L884P JAK2 is colored in yellow or orange). Differences of the total interactions (enthalpies) for the WT and L884P JAK2 complexes are illustrated in panel B. Comparison of the non-polar and the polar part contributions for the WT (blue) and L884P (yellow) JAK2 complexes are illustrated in panels C and D. Comparison of the RMSFs of the WT (green) and L884P (colorful)/CHZ868 complexes is shown in panel E. (the individual pictures of Fig. 6A~E correspond to Figure S8A~E in Figure S8 of supplementary information).

### Both Non-polar and Polar Interactions are Important to Drug Resistance

As summarized in Table 2, the binding free energies (∆*G*_bind_) and the corresponding components were calculated by the MM/GBSA approach based on the conventional MD trajectories for the WT and L884P JAK2s in complex with BBT594 and CHZ868. The predicted enthalpies (Δ*E*_enthalpy_) for L884P/BBT594 and L884P/CHZ868 are −49.60 and −53.41 kcal/mol, respectively, which are both higher than those for the corresponding WT systems (−52.10 and −54.27 kcal/mol) and are consistent with the experimental data. The non-polar contributions (Δ*E*_vdw_ + Δ*G*_SA_) for the WT/BBT594 and L884P/BBT594 complexes are −79.11 and −77.95 kcal/mol, respectively, and those for the WT/CHZ868 and L884P/CHZ868 complexes are −68.81 and −67.73 kcal/mol, respectively, suggesting that the decrease of the non-polar contributions caused by the L884P mutation accounts for the drug resistance of the two Type-II inhibitors.

The polar contribution (Δ*E*_ele_ + Δ*G*_GB_) for the WT/BBT594 and L884P/BBT594 complexes are 28.36 and 27.09 kcal/mol, respectively, and those for the WT/CHZ868 and L884P/CHZ868 complexes are almost identical (14.54 and 14.33 kcal/mol). That is to say, the L884P mutation weakens the polar contribution to the binding of BBT594, but has no obvious impact on the polar contribution to the binding of CHZ868. Therefore, it can be concluded that both the polar and non-polar interactions are vital factors for the resistance of JAK2 to BBT594, while only the non-polar interaction is important to the resistance of JAK2 to CHZ868.

From the per residue decomposition analysis, as shown in Table S2, we can identify the key residues for the ligands binding, which are mainly located in the hinge region, DFG motif, β-strand, and αC-helix of JAK2. To be more detailed, Fig. 5A (Figure S7A) exhibits that, in the WT and L884P systems, urea-CO of BBT594 forms a H-bond with Asp994 of the DFG-out motif (−3.20 *versus* −2.80 kcal/mol) and charge-reinforced H-bonds with the conserved αC-helix residue Glu898 (0.78 *versus* 2.62 kcal/mol). Besides, two more H-bonds are formed between amino-pyrimidine of BBT594 and Leu932 (−3.40 *versus* −2.80 kcal/mol), as well as the backbone-CO of His974 with the protonated N-methylpiperazine (−1.84 *versus* −1.72 kcal/mol). Apparently, the H-bond interactions become weaker after Leu884 in JAK2 is mutated to Pro884, suggesting that the H-bonds, in addition to stabilizing the ligand in the binding pocket, also play an important role in determining drug resistance.

Moreover, the difference of other non-H-bond interactions cannot be neglected (Table S2). For example, Tyr931 (−3.02 *versus* −0.20 kcal/mol), Leu902 (−3.22 *versus* −2.74 kcal/mol) and Tyr972 (−3.28 *versus* −2.64 kcal/mol) form stronger interactions with BBT594 in the WT system than those in the L884P system. As shown in Figs 5B (S7B), 5C (S7V) and 5D (S7D), the attenuation of the van der Waals interaction of Tyr931 and the increase of the adverse polar solvation energy of Glu898 are the most important contributors to the decrease of the binding of BBT594 to the L884P JAK2. The change of the ligand-residue interaction between the WT and mutated systems can be explained by the conformational changes of the binding pocket induced by the L884P mutation in JAK2. According to the superposed structures of the binding pockets shown in Figs 5A (S7A), we can observe that the β-strand, and αC-helix of the mutated JAK2 (blue) exhibit obviously upward movement, which undoubtedly affects the interactions between BBT594 and the residues of the αC-helix (Glu898 and Leu902). Moreover, several residues located in other part of the binding pocket in the mutated JAK2, such as Tyr931, Asp994, and Tyr972, also alter their conformations and locations.

As for CHZ868, the above mentioned energy differences of the key residues between WT and L884P still exist (Figs 6B or S8B), but the difference is relatively smaller (−1.62 *versus* −1.22 kcal/mol for Glu898, −3.14 *versus* −2.86 kcal/mol for Val911, −1.28 *versus* −1.04 for Leu905 and −1.22 *versus* −1.00 for Ile901), suggesting the stronger anti-resistance capability of CHZ868 to the L884P mutation. Moreover, the residue-ligand interactions illustrated in Figs 6A (S8A) and 6B (S8B) further confirm the dominant responsibility of the hydrophobic interactions for drug resistance in the CHZ868 systems. In contrast to the bulky tail (1-Methyl-4-[2-(trifluoromethyl)penzyl] methyl]-piperazine) of BBT594, the small size tail (1,3-difluorobenzene moiety) of CHZ868 intends to form more favorable interaction (H-bond or hydrophobic interactions) with the residues located in the allosteric pocket (−0.04 *versus* −3.16 kcal/mol for Lys882, 0.78 *versus* −1.22 kcal/mol for Glu898 and −3.20 *versus* −5.18 kcal/mol for Asp994, Table S2). According to Figs 6A (S8A), compared with the obvious conformational changes between the WT and L884P/ BBT594 systems (Figs 5A and S7A), the above mentioned stronger interactions in the CHZ868 system can more effectively hinder the movement of the β-strand and αC-helix (even still exist) induced by the L884P mutation.

## Conclusion

In summary, we have successfully characterized the bindings of BBT594 and CHZ868 to the WT JAK2 and its drug resistant variant (L884P), both structurally and energetically, by combining multiple molecular modeling methodologies, such as conventional MD simulations, US simulations, and MM/GBSA free energy calculations and decompositions. According to the US simulations, we can observe that the L884P mutation enhances the flexibility of the allosteric pocket, especially the β3-strand, αC-helix and DFG motif, which was supported by the increased conformational entropy (−TΔ*S*) and RMSFs. Quantitatively, the energy decomposition analyses suggest that interactions of the majority of the key residues surrounding the binding pocket to the ligands are impaired after Leu884 is mutated to Pro884, and among them, the attenuation of the van der Waals interaction of Tyr931 and the increase of the adverse polar solvation energy of Glu898 should be the most important contributors to the decrease of the BBT594 binding to the mutated JAK2. Moreover, the moderate influence of the mutation on the CHZ868/JAK2 system can be explained by the smaller size of the drug tail which forms stronger interactions with some residues in the allosteric pocket of JAK2. Therefore, the optimization of the tail moiety, located in the allosteric pocket of JAK2 kinase, of Type-II inhibitors should be emphasized in the future study.

## Materials and Methods

### Systems Setup and Molecular Dynamics (MD) simulations

The co-crystallized structure of the WT JAK2 in complex with BBT594 was downloaded from RCSB Protein Data Bank^37^ (PDB code: 3UGC) and used as the initial structure for computational simulations. The missing residues, such as the A-loop (Val1000-Pro1013), were added by the *loop* module in SYBYL-X1.0^38^, followed by conformational adjustment to relieve the unfavorable interaction of the newly added/repaired fragments with the surroundings. The protonation states of the residues in JAK2 were determined by PROPKA 3.1^39^. Considering the similar structure scaffold between CHZ868 and BBT594, the bound-state WT/CHZ868 was predicted by docking CHZ868 into the binding pocket of the WT JAK2 (3UGC) using the *Glide* module in Schrodinger 2015^40^. As shown in Figure S9, the core structures of BBT594 and CHZ868 are well superposed (RMSD = 1.093 Å). The L884P mutations in BBT594 and CHZ868 JAK2 systems were accomplished by the *biopolymer* module in SYBYL-X1.0.

The two Type-II inhibitors were firstly optimized by the Hartree-Fock (HF) method at 6–31 G* level of theory implemented in Gaussian 09^41^, and the same level of theory was employed for the electrostatic potential calculation as well. After that, the restrained electrostatic potential technique (RESP) was used to fit the atomic partial charges of the inhibitors. The AMBER14SB force field^42^ and the general AMBER force field (gaff)^43^ were employed for the proteins and inhibitors, respectively. Each complex was immersed into a cubic TIP3P water box^44^ with 10 Å extension of water molecules away from each face of the complex, and 1 Cl^−^ was added to neutralize the redundant charges of each ligand-receptor complex.

Prior to MD simulation, the constrained hydrogen atoms, water molecules and ions, and the backbone atoms of protein in each system (5 kcal/(mol·Å^2^)) were sequentially relaxed and then optimized by 1000 cycles of steepest descent minimization and 4000 cycles of conjugate gradient energy minimization. Then, the whole system was optimized by 10000 cycles of minimization without any restraint. After 50 ps heating-up stage (from 0 to 300 K in the NVT ensemble) and 50 ps equilibration stage (in the NPT ensemble at *P* = 1 atm and *T* = 300 K), 30 ns conventional MD simulation in the NPT ensemble (*T* = 300 K and *P* = 1 atm) was performed for each system by the *pmemd* module in AMBER14 package^45^, where the time step was set to 2 fs and the MD trajectory was saved every 10 ps. The Particle Mesh Ewald (PME) technique^46^ was employed to calculate the long-range electrostatics interactions with a direct-space cutoff of 8 Å, and the same threshold value was also used for truncation of the Lennard-Jones potentials. Hydrogen atoms involved in covalent bonds were constrained by the SHAKE algorithm^47^.

### Umbrella Sampling (US) Simulations

US is one of the most classical enhanced sampling methodologies to characterize the dissociation pathway of an inhibitor from its target^48^. By imposing biasing potentials on the reaction coordinate (RC), US can derive the system from one thermodynamic state to another that may be divided by high energy barriers, therefore making the method very helpful in interpreting drug-target interactions such as the mechanisms of drug resistance and drug selectivity^36, 48–50^. To determine the favorable unbinding pathways of the two Type-II kinase inhibitors that occupy both the ATP-binding and allosteric pockets of JAK2, herein, the inhibitors were dragged out along two channels (the detailed analysis can be found in the following section). The directions of the RCs along these two channels were identified by using the Caver 2.0 module in PYMOL software^51^, where the largest pocket direction was set as the initial unbinding direction for each US simulation. In WT JAK2/BBT594 system, the reaction coordinate (RC) along the allosteric channel is the distance between the carbon atom (CB) of Met 865 in receptor and the carbon atom (C24) of BBT594 (L884P JAK2/BBT594: carbon atom (CE) of Met 865, carbon atom (C6) of BBT594; WT JAK2/CHZ868: sulfur atom (SD) of Met 865, carbon atom (C26) of CHZ868; L884P JAK2/CHZ868: sulfur atom (SD) of Met865, carbon atom (C26) of CHZ868), and the RC along the ATP channel is the distance between the carbon atom (CD1) of Tyr972 in receptor and the carbon atom (C11) of BBT594. (L884P JAK2/BBT594: carbon atom (CZ) of Tyr 972, carbon atom (C18) of BBT594; WT JAK2/CHZ868: carbon atom (CD2) of Tyr 972, carbon atom (C4) of CHZ868; L884P JAK2/CHZ868: carbon atom (CE2) of Tyr 972, carbon atom (C4) of CHZ868) (Figure S10) The US simulations were operated across 41 continuous windows with 0.5 Å in length for each, which means a total of 20 Å of the RC extending away from the original position (bound-state) of each inhibitor. The harmonic potential was added according to ${\mu }_{i}=\frac{1}{2}{k}_{i}{(r-{r}_{i})}^{2}$, where *k*_*i*_ represents the restraint potential elastic constant and was set to 5 kcal/mol·Å^2^ in each window, and *r* and *r*_*i*_ denote the current value and the starting value of the ligand RCs in window *i*, respectively. For each system, 10 ns US simulations were performed for each window to sufficiently converge the potential of the mean force (PMF). The weighted histogram analysis method (WHAM)^52, 53^ was employed to estimate the PMF curve along the RC by reconstructing the biased probability distribution of each window to the normal one. As shown in Figures S3 and S4 of the Supporting Information, the PMF curves derived from the last 4 ns US simulations were convergent and were finally chosen for detailed analyses.

### Single Trajectory Based MM/GBSA Calculations

The Molecular Mechanics/Generalized Born Surface Area (MM/GBSA) method^54–60^, widely used in elucidating drug resistance mechanisms^61, 62^, was employed to estimate the binding free energies for the WT/BBT594, L884P/BBT594, WT/CHZ868 and L884P/CHZ868 systems based on the 2800 snapshots extracted from the 2~30 ns MD trajectories (Figure S1 and S2). According to Eq. 1, the total binding free energy (Δ*G*_bind_) can be subdivided into several terms, including the van der Waals interaction (Δ*E*_vdW_), the electrostatic interaction (Δ*E*_ele_), the polar (Δ*G*_GB_) and non-polar (Δ*G*_SA_) components of the solvation free energy (Δ*G*_solvation_), and the conformational entropy upon ligand binding (−*T*Δ*S*), which facilitate to ascertain the crucial factor to govern drug resistance^56, 61–63^.

1 $$\begin{array}{rcl}{\rm{\Delta }}{G}_{bind} & = & {G}_{com}-({G}_{rec}+{G}_{lig})\\  & = & {\rm{\Delta }}H+{\rm{\Delta }}{G}_{solvation}-T{\rm{\Delta }}S\\  & = & {\rm{\Delta }}{E}_{int}+{\rm{\Delta }}{E}_{ele}+{\rm{\Delta }}{E}_{vdW}+{\rm{\Delta }}{G}_{GB}+{\rm{\Delta }}{G}_{SA}-T{\rm{\Delta }}S\end{array}$$

The *sander* module in Amber14 was used to calculate Δ*H* (including Δ*E*_*int*_, Δ*E*_*vdW*_ and Δ*E*_*ele*_), where Δ*E*_*int*_, change of the intramolecular energies upon ligand binding, can be canceled out due to the use of the single trajectory strategy. The polar part of the solvation energy (Δ*G*_GB_) was calculated by using the GB model developed by Onufriev *et al*. (GB^OBC1^, *igb* = 2)^64^, which performed better than the other GB models implemented in Amber^55^. The solute (*ε*_in_) and solvent (*ε*_out_) dielectric constants were set to 1 and 80, respectively^65^. The non-polar part of the solvation energy (Δ*G*_SA_) was estimated by the change of the solvent-accessible surface areas (ΔSASA) through the LCPO algorithm: Δ*G*_*SA*_ = *γ* × ΔSASA + *β*, where *γ* and *β* were set to 0.0072 kcal/(mol·Å^2^) and 0 kcal/(mol·Å^2^), respectively. The conformational entropy (−*T*Δ*S*) was calculated by normal mode analysis (NMA) implemented in the *nmode* module of AMBER14^62, 66, 67^. To save computational cost, 92 snapshots evenly extracted from the 2~30 ns equilibrated MD trajectories were used for the entropy calculations (Table S1).

Binding free energy decomposition supported by *MMPBSA*.*py* script^68^ was then used to identify the residues important to drug resistance. Per ligand-residue interaction was calculated according to Δ*G*_ligand-residue_ = Δ*E*_vdW_ + Δ*E*_ele_ + ∆*G*_GB_ + ∆*G*_SA_. Except for ∆*G*_SA_, which was calculated by the ICOSA algorithm^69^, the other terms were calculated based on the same parameters used in the above MM/GBSA calculations.

## Electronic supplementary material

Supplementary Information
