# Supplementary material for: How Does the L884P Mutation Confer Resistance to Type-II Inhibitors of JAK2 Kinase: A Comprehensive Molecular Modeling Study
Source: Sci Rep. 2017 Aug 22;7:9088. doi: 10.1038/s41598-017-09586-3 (PMC5567357; doi:10.1038/s41598-017-09586-3)
Supplement: Supplementary file 1 — Supplementary Information [file 41598_2017_9586_MOESM1_ESM.pdf]

Supplementary Information

How Does the L884P Mutation Confer Resistance to Type-II Inhibitors of JAK2 Kinase: A Comprehensive Molecular Modeling Study

Xiaotian Kong,[a,b] Huiyong Sun,[b] Peichen Pan,[b] Dan Li,[b] Feng Zhu,[b] Shan Chang,[c] Lei Xu,[c] Youyong Li,[a]* Tingjun Hou[a,b]*

[a]Institute of Functional Nano and Soft Materials (FUNSOM), Soochow University, Suzhou, Jiangsu 215123, P. R. China.

[b]College of Pharmaceutical Sciences, Zhejiang University, Hangzhou, Zhejiang 310058, P. R. China.

[c]Institute of Bioinformatics and Medical Engineering, School of Electrical and Information Engineering, Jiangsu University of Technology, Changzhou 213001, China

The authors declare no competing financial interest.

Corresponding authors:

Tingjun Hou

E-mail: tingjunhou@zju.edu.cn

Youyong Li

E-mail: yyli@suda.edu.cn

Table S1. Conformational entropies calculated based on 23 blocks with 5 snapshots/blocks.

	**WT-BBT594**	**L884P-BBT594**	**WT-CHZ868**	**L884P-CHZ868**
1	-31.34	-30.25	-21.69	-26.85
2	-28.58	-28.35	-28.32	-23.21
3	-24.36	-29.93	-30.75	-20.22
4	-28.18	-31.33	-28.07	-27.37
5	-28.14	-27.53	-23.17	-25.86
6	-18.13	-28.22	-22.39	-26.55
7	-28.01	-28.11	-18.80	-28.29
8	-27.88	-30.21	-22.47	-27.78
9	-25.02	-22.30	-21.41	-27.90
10	-29.99	-21.69	-25.10	-24.14
11	-25.26	-25.35	-26.99	-20.90
12	-30.80	-27.49	-30.87	-26.96
13	-28.99	-30.29	-25.79	-34.47
14	-32.47	-32.03	-26.09	-23.79
15	-16.02	-24.27	-27.09	-24.15
16	-27.12	-17.22	-25.76	-23.17
17	-31.14	-28.76	-20.63	-20.79
18	-32.13	-33.18	-24.61	-34.90
19	-23.95	-28.66	-26.30	-33.04
20	-20.70	-32.58	-22.74	-32.20
21	-24.96	-31.13	-25.37	-24.88
22	-27.44	-24.50	-27.55	-23.69
23	-23.39	-28.69	-27.93	-25.18
average	-26.70±1.24	-27.90±1.45	-25.18±3.11	-26.32±2.16

Table S2. Free energy contributions of the key residues surrounding the binding pocket to the bindings of the inhibitors (kcal/mol)

	**WT-BBT594**	**L884P-BBT594**	**WT-CHZ868**	**L884P-CHZ868**
LEU855	-2.22	-1.88	-2.02	-2.18
GLY856	-0.02	-0.02	-0.02	-0.02
VAL863	-1.94	-2.08	-2.38	-2.46
ALA880	-2.28	-2.28	-2.52	-2.5
VAL881	-0.28	-0.26	-0.98	-0.98
LYS882	-0.04	-0.14	-3.16	-3.04
GLU898	0.78	2.62	-1.62	-1.22
ILE901	-1.88	-2.7	-1.22	-1
LEU902	-3.22	-2.74	-2.44	-2.6
LEU905	-2.22	-1.96	-1.28	-1.04
ILE910	-1.42	-1.38	-0.92	-0.92
VAL911	-3.2	-3.08	-3.14	-2.86
LEU927	-1.4	-1.26	-1.56	-1.38
MET929	-4.1	-4.78	-4.3	-4.32
GLU930	-1.72	-2.46	-1.22	-1.1
TYR931	-3.02	-0.2	-4.2	-4.06
LEU932	-3.4	-2.8	-4.44	-4.38
GLY935	-1.42	-1.3	-1.4	-1.54
TYR972	-3.28	-2.64	-0.04	-0.02
HIS974	-1.84	-1.72	-0.54	-0.48
LEU983	-2.68	-2.64	-3.82	-3.8
ILE992	-1.02	-1.18	-1.2	-1.22
GLY993	-4.16	-3.88	-3.44	-3.54
ASP994	-3.2	-2.82	-5.18	-5.18

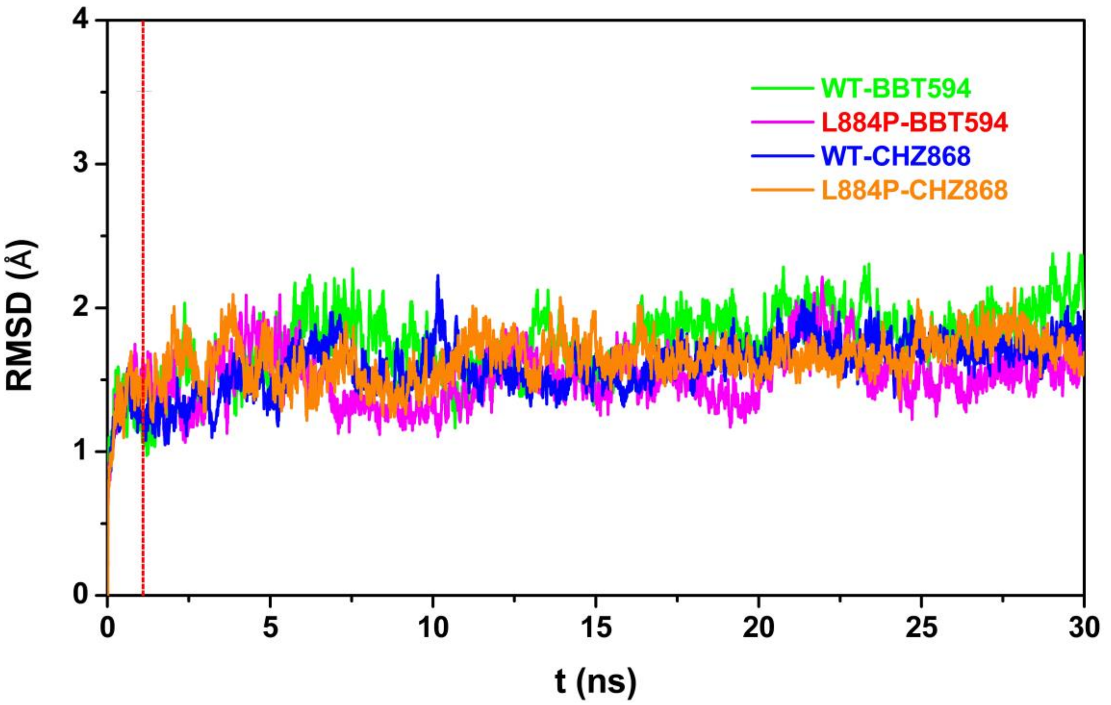

Figure S1. The root-mean-square deviations (RMSDs) of protein-ligand complexes of the studied systems.

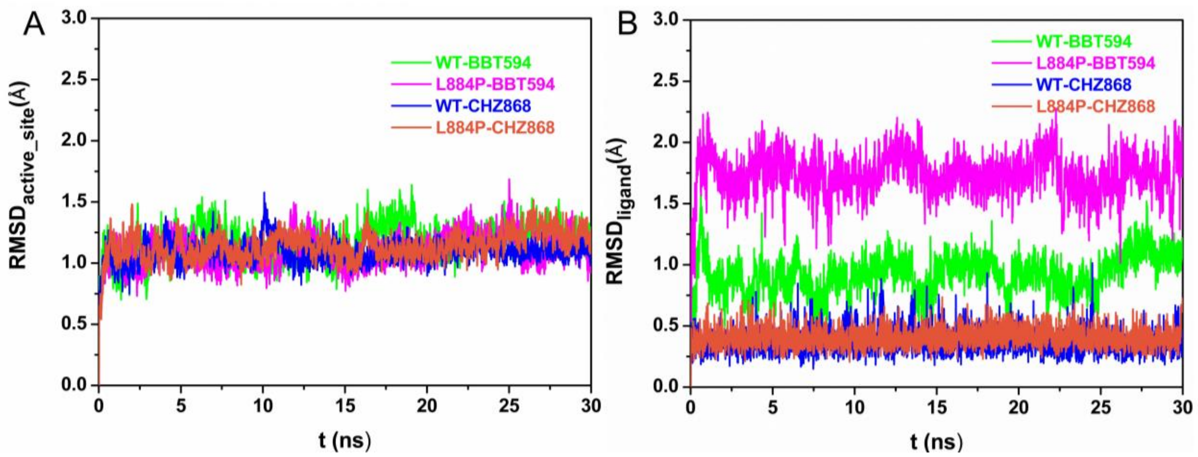

Figure S2. The root-mean-square deviations (RMSDs) of (A) the residues that shape the binding cavity (active site), (B) the ligand of the studied systems.

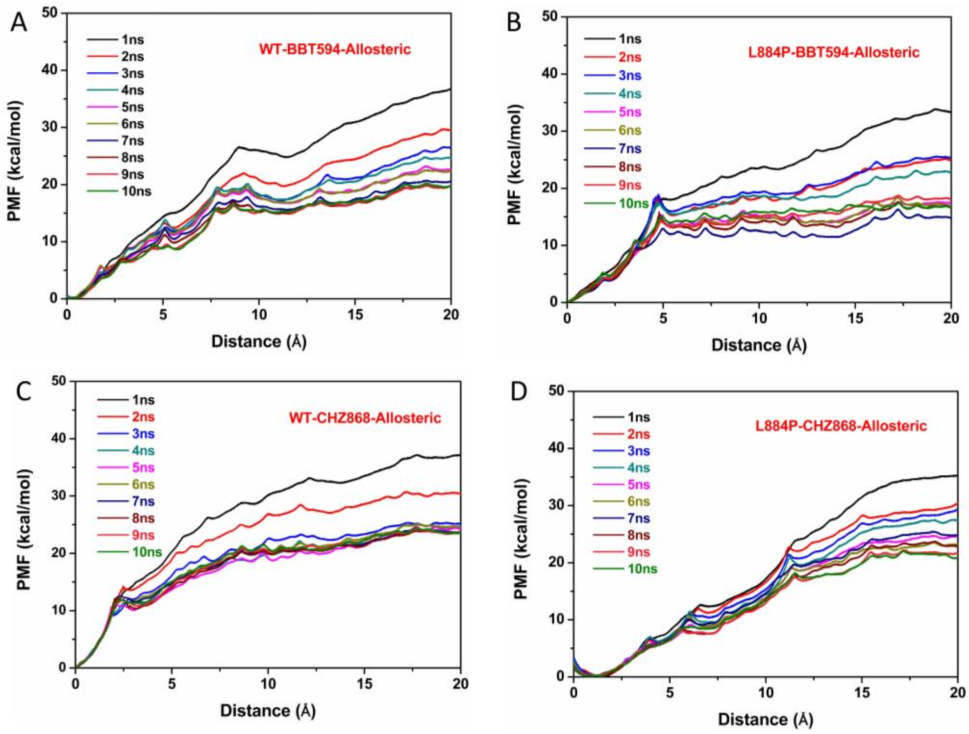

Figure S3. Convergence of the simulated systems along allosteric channel.

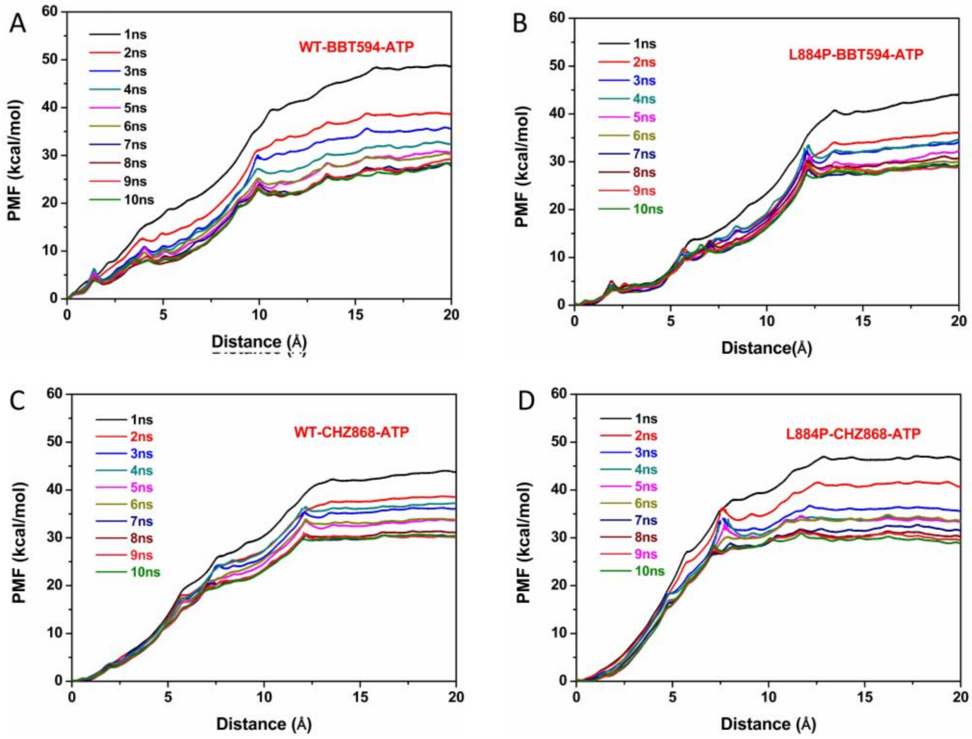

Figure S4. Convergence of the simulated systems along ATP channel.

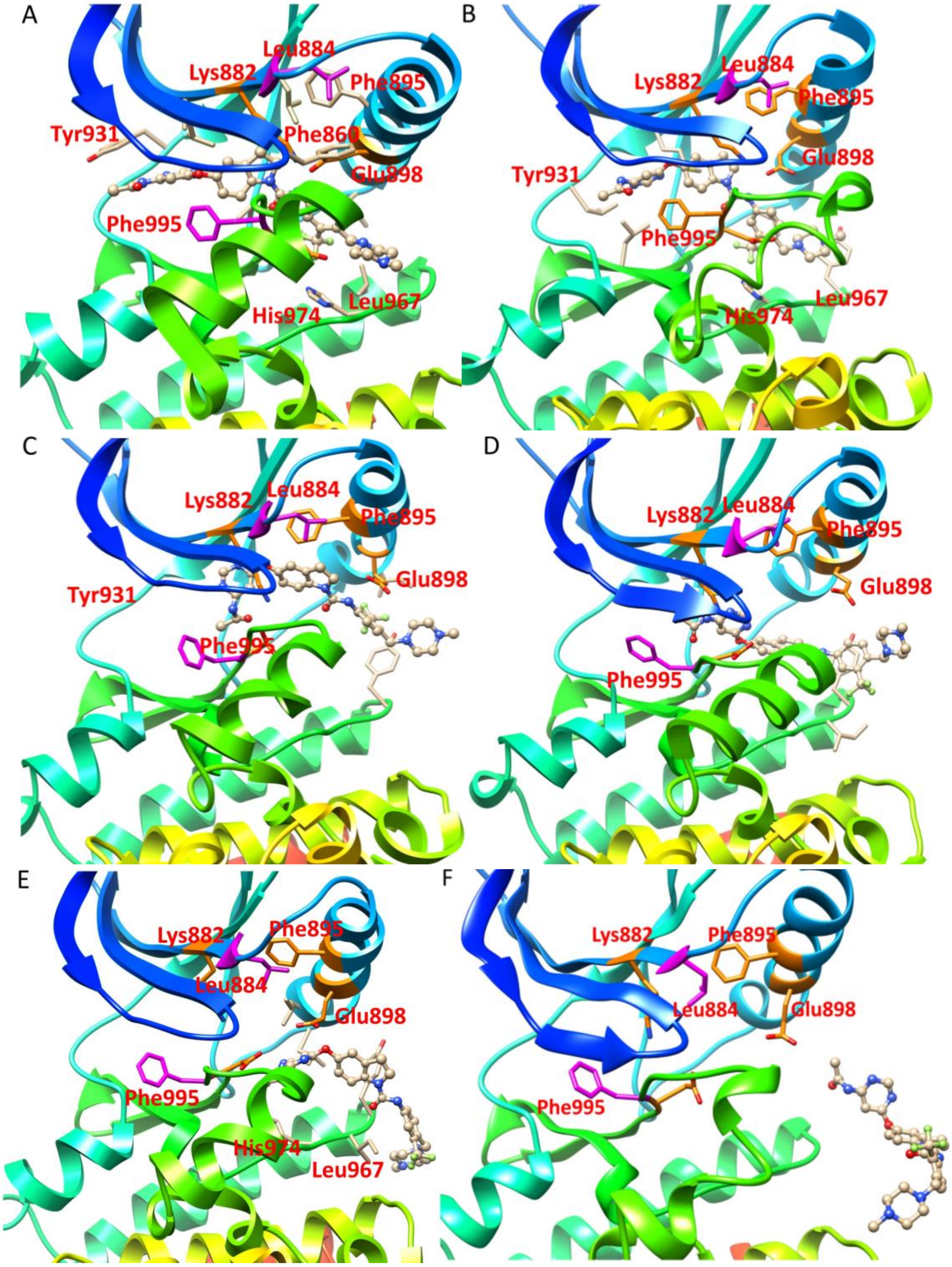
A
Leu884
Lys882
Phe895
Tyr931
Phe860
Glu898
Phe995
His974
Leu967
B
Lys882
Leu884
Phe895
Glu898
Tyr931
Phe995
Leu967
His974
C
Lys882
Leu884
Phe895
Glu898
Tyr931
Phe995
D
Lys882
Leu884
Phe895
Glu898
Phe995
E
Lys882
Phe895
Leu884
Glu898
Phe995
His974
Leu967
F
Lys882
Phe895
Leu884
Glu898
Phe995

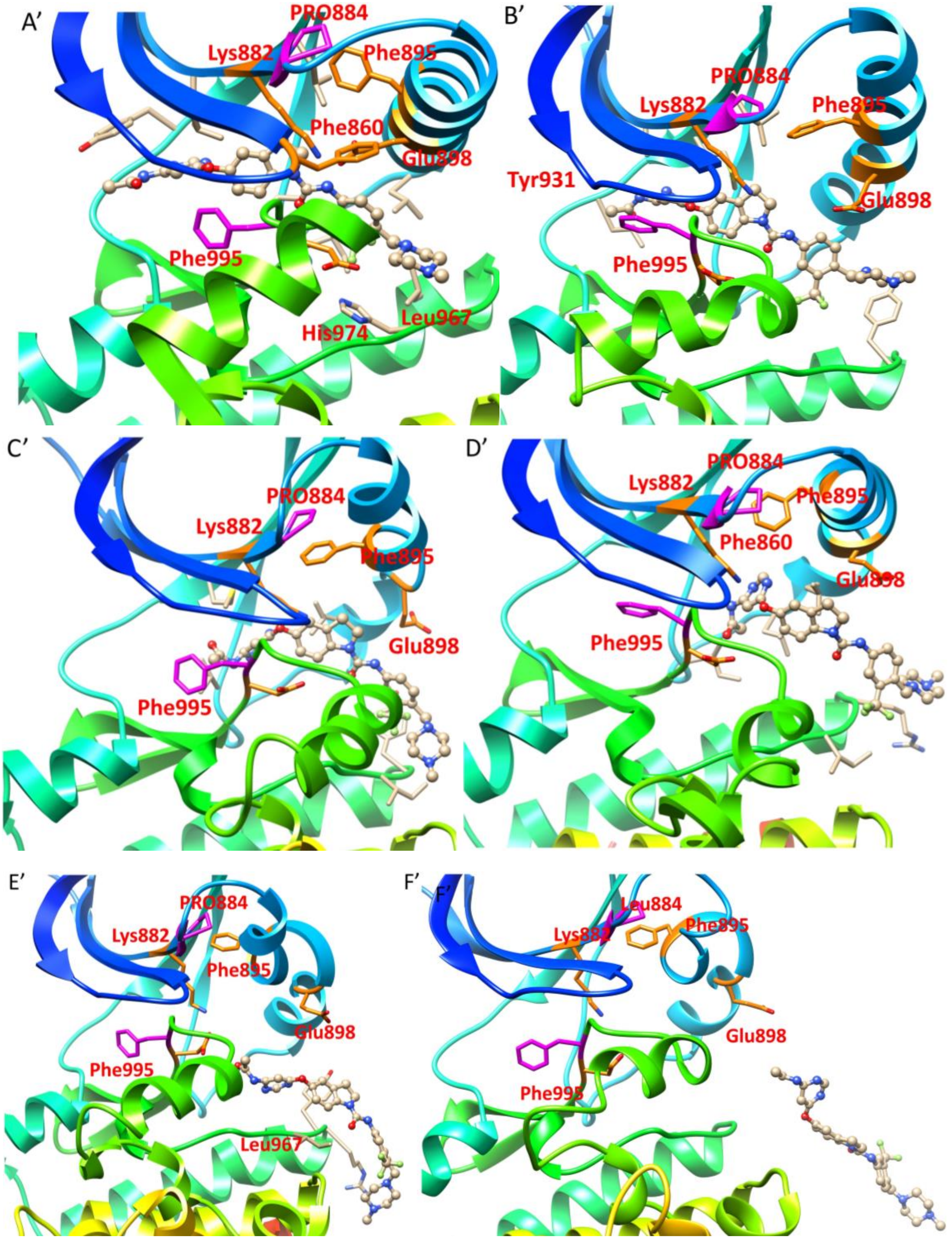

Figure S5. Unbinding processes of Type-II inhibitor BBT594 dissociating from the binding sites of the WT (panels A~F) and L884P (panels A'~F') JAK2 along the allosteric channel. (Figures A~F and A’~F’ correspond to panels 3A~3F and 3A’~3F’ in Figure 3)

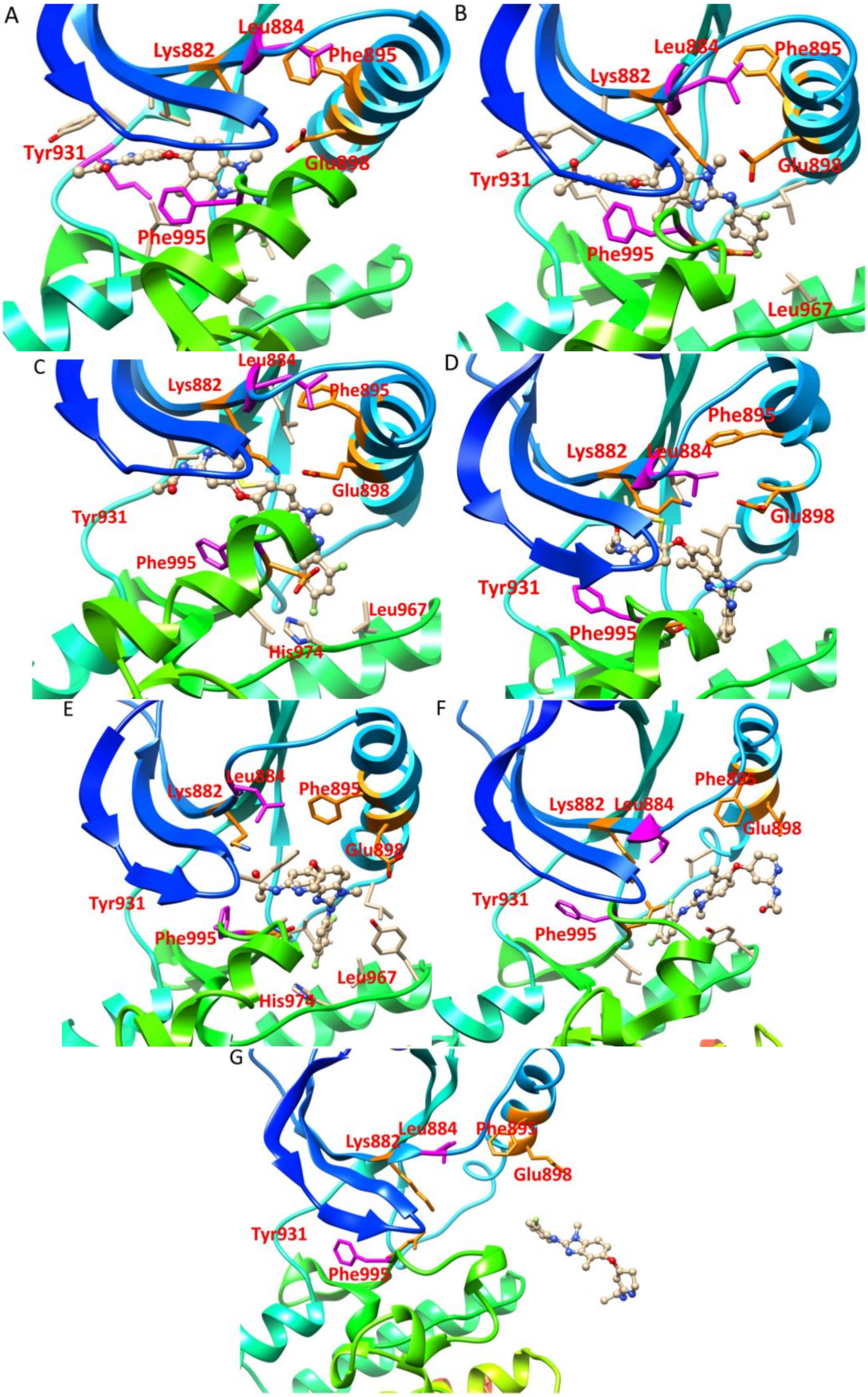

A
Leu884
Lys882
Phe895
Tyr931
Glu898
Phe995
B
Leu884
Lys882
Phe895
Tyr931
Glu898
Phe995
Leu967
C
Leu884
Lys882
Phe895
Glu898
Tyr931
Phe995
Leu967
His974
D
Phe895
Lys882
Leu884
Glu898
Tyr931
Phe995
E
Leu884
Lys882
Phe895
Glu898
Tyr931
Phe995
Leu967
His974
F
Phe895
Lys882
Leu884
Glu898
Tyr931
Phe995
G
Lys882
Leu884
Phe895
Glu898
Tyr931
Phe995

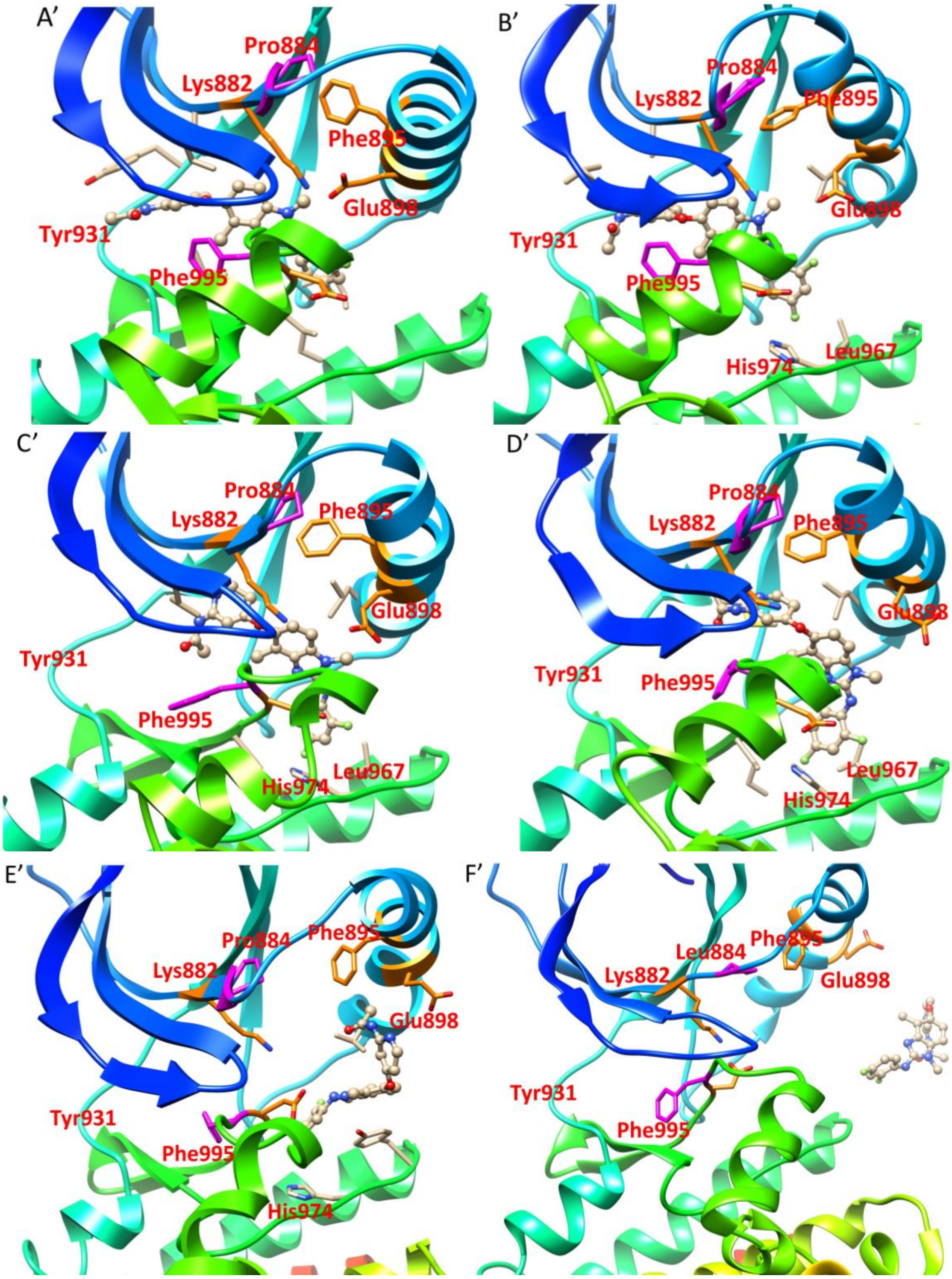

Figure S6. Unbinding processes of Type-II inhibitor CHZ868 dissociating from the binding sites of the WT (panels A~G) and L884P (panels A'~F') JAK2 along the allosteric channel. (Figures A~G and A'~F' correspond to panels 4A~4G and 3A'~3F' in Figure 4.)

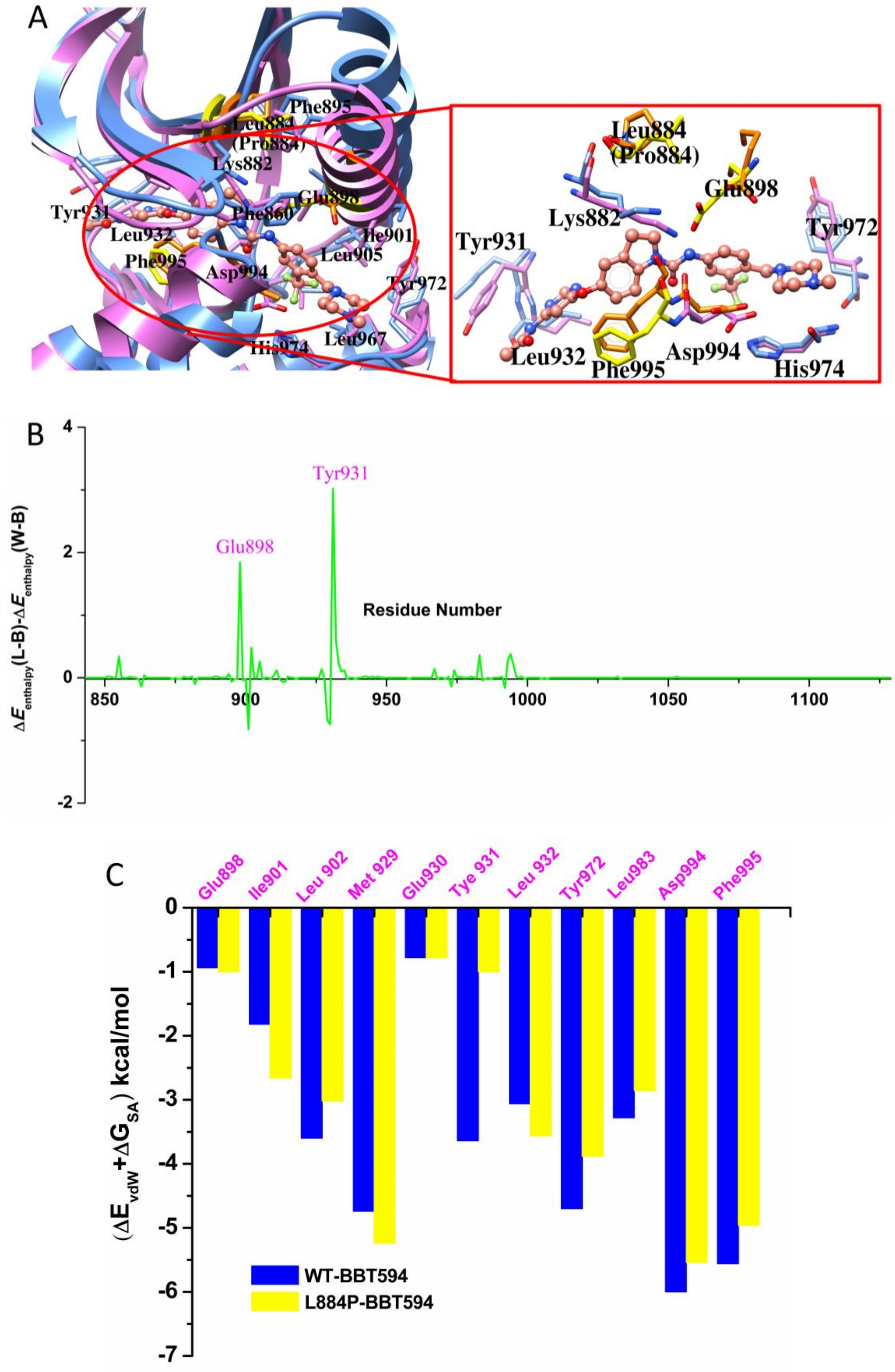
A
Phe895
Leu884
(Pro884)
Lys882
Glu898
Tyr931
Phe860
Ile901
Leu932
Leu905
Phe995
Asp994
Tyr972
His974
Leu967
Leu884
(Pro884)
Glu898
Lys882
Tyr972
Tyr931
Leu932
Phe995
Asp994
His974
B
$\Delta E_{enthalpy}$(L-B)-$\Delta E_{enthalpy}$(W-B)
Tyr931
Glu898
Residue Number
4
2
0
-2
850
900
950
1000
1050
1100
C
Glu898
Ile901
Leu 902
Met 929
Glu930
Tye 931
Leu 932
Tyr972
Leu983
Asp994
Phe995
(ΔE_{vdW}+ΔG_{SA}) kcal/mol
0
-1
-2
-3
-4
-5
-6
-7
WT-BBT594
L884P-BBT594

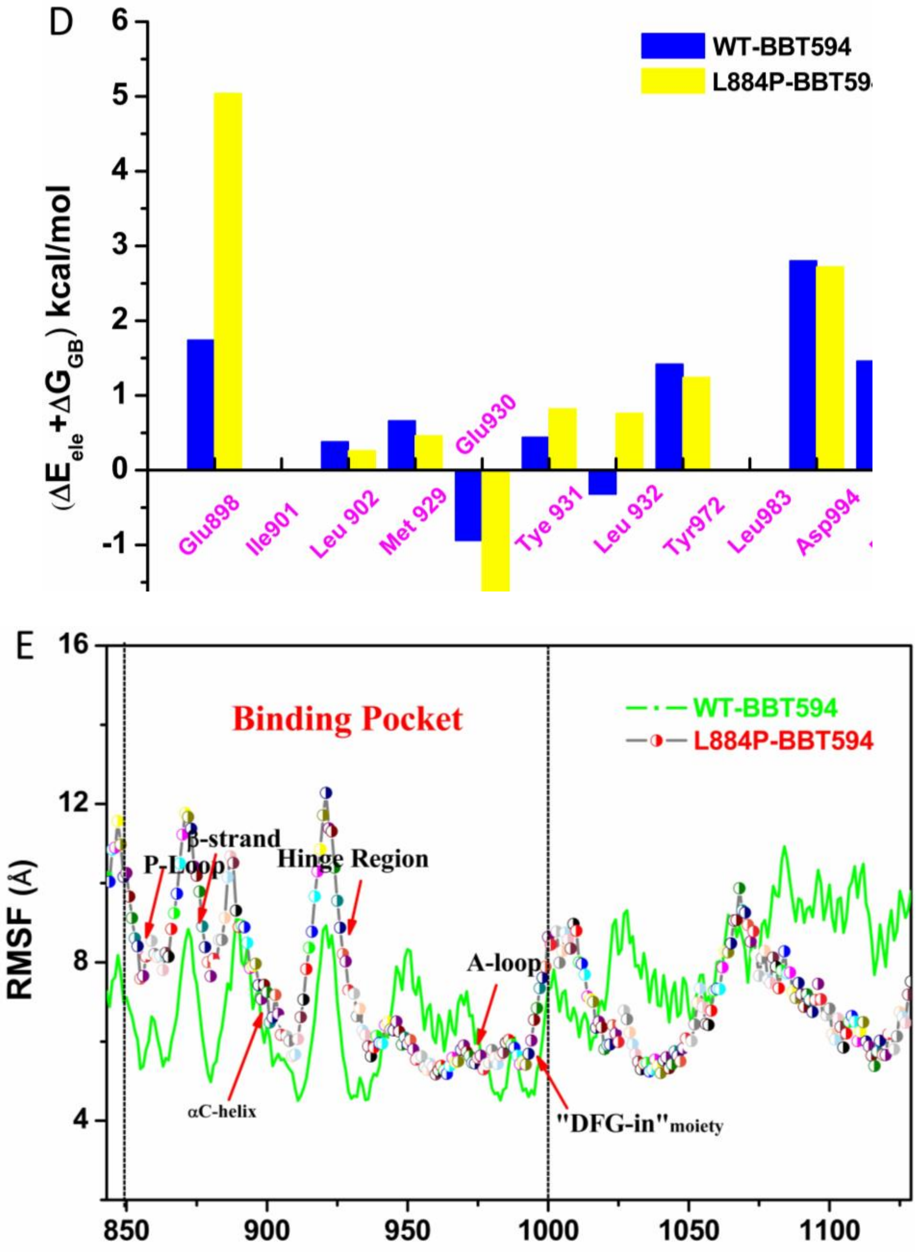

Figure S7. Figure A~E correspond to Figure 5A~5E in Figure 5.

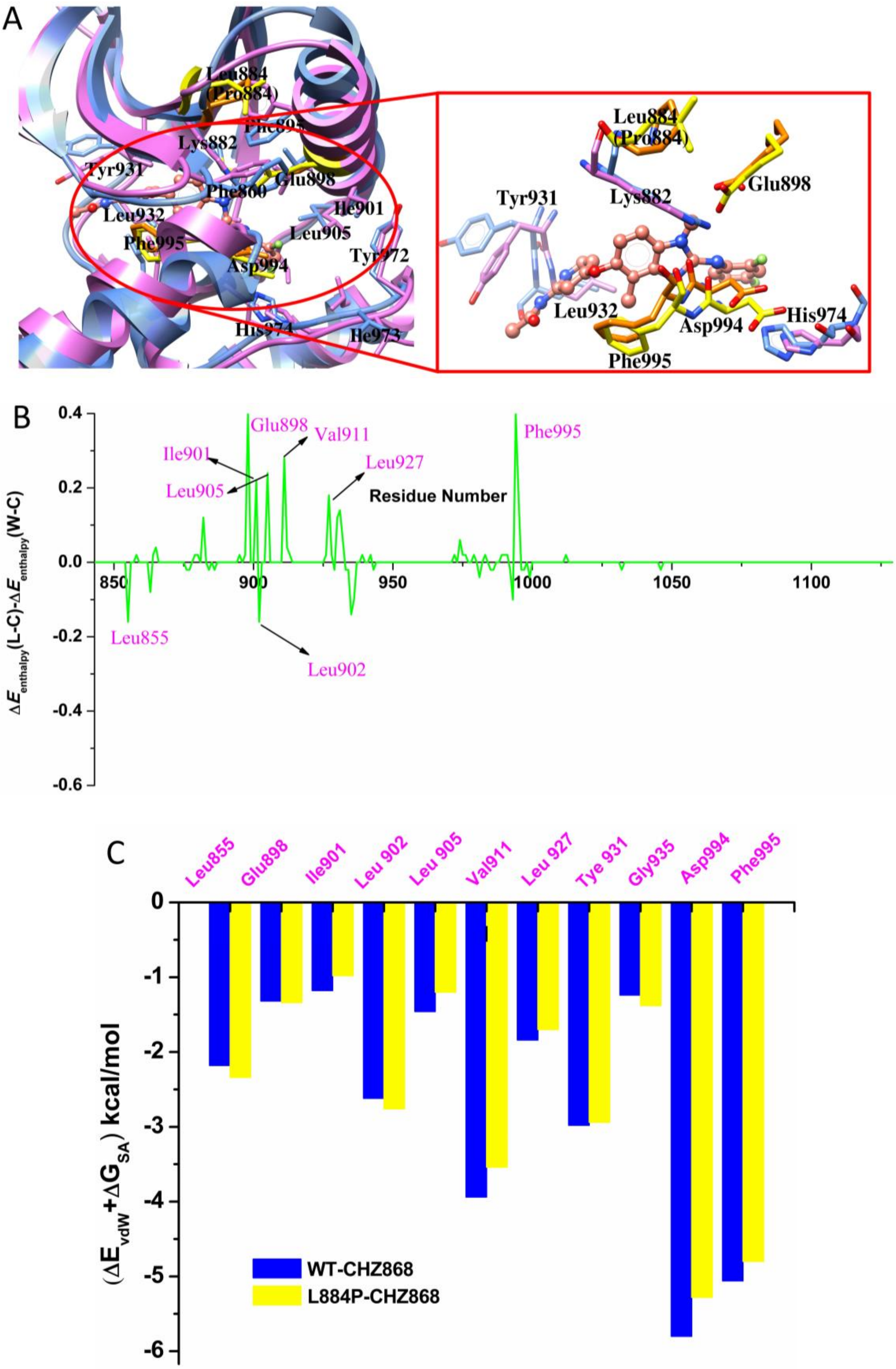
A
Leu884
(Pro884)
Phe895
Lys882
Tyr931
Glu898
Phe860
Ile901
Leu932
Leu905
Phe995
Tyr972
Asp994
His974
Ile973
Leu884
(Pro884)
Glu898
Tyr931
Lys882
Leu932
Asp994
His974
Phe995
B
$\Delta E_{enthalpy}$(L-C)-$\Delta E_{enthalpy}$(W-C)
Residue Number
Glu898
Val911
Phe995
Ile901
Leu927
Leu905
Leu855
Leu902
0.4
0.2
0.0
-0.2
-0.4
-0.6
850
900
950
1000
1050
1100
C
Leu855
Glu898
Ile901
Leu 902
Leu 905
Val911
Leu 927
Tye 931
Gly935
Asp994
Phe995
(ΔE_{vdW}+ΔG_{SA}) kcal/mol
0
-1
-2
-3
-4
-5
-6
WT-CHZ868
L884P-CHZ868

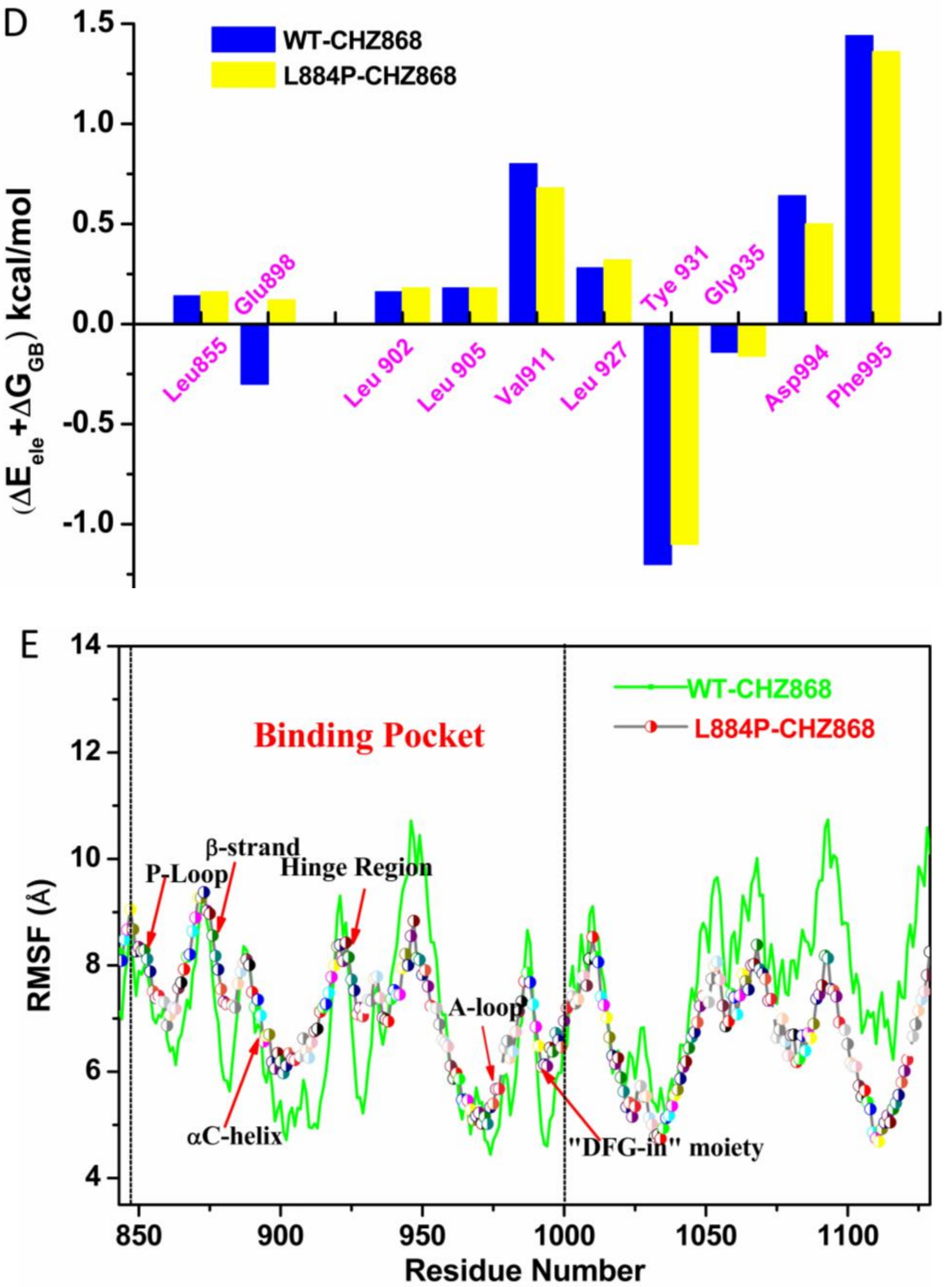

Figure S8. Figures A~E correspond to panels Figure 6A~6E in Figure 6.

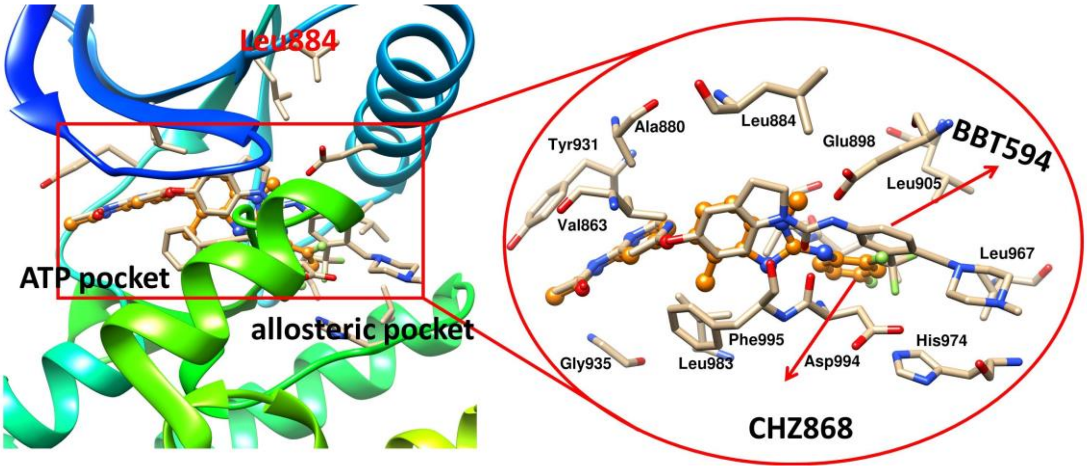

Figure S9. Structural superimposition of the co-crystallized structure of WT/BBT594 (PDB entry: 3UGC) and the structure of JAK2/CHZ868 predicted by the *Glide* docking.

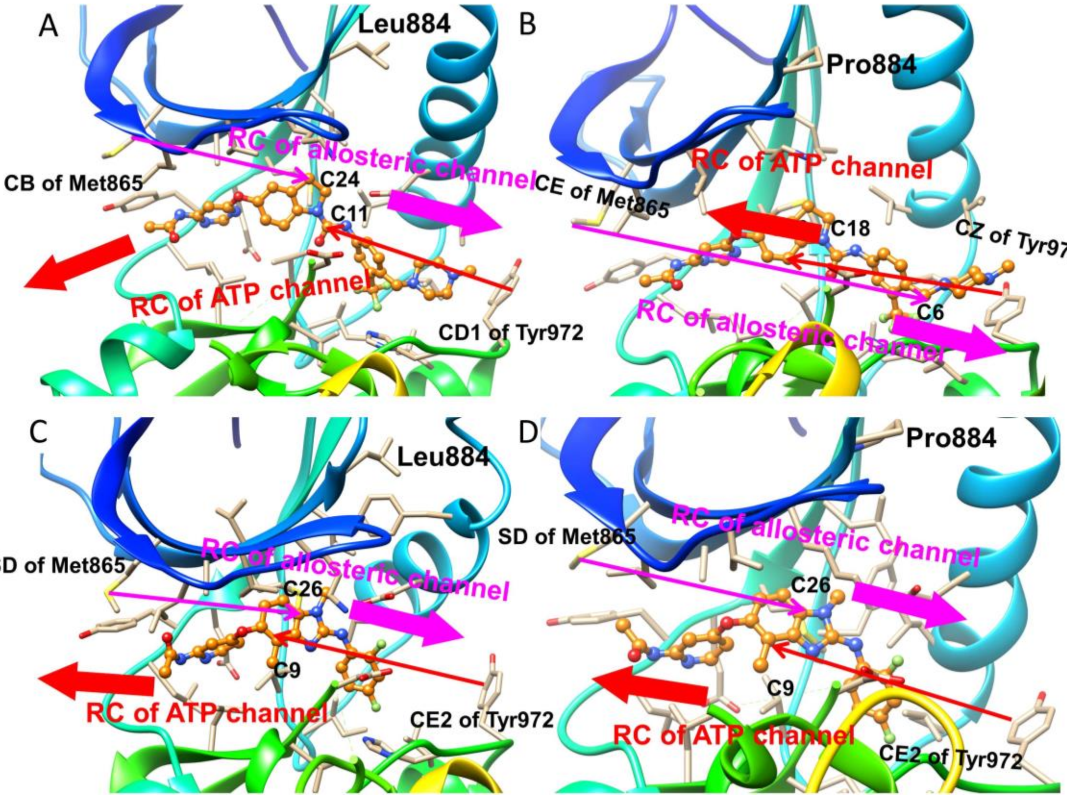

Figure S10. The RCs through ATP and allosteric channels of (A) WT JAK2/BBT594, (B) L884P JAK2/BBT594, (C) WT JAK2/CHZ868, (D) L884P JAK2/CHZ868 systems.
